# Role of Sphingomyelin Synthase in Controlling the Antimicrobial Activity of Neutrophils against *Cryptococcus neoformans*

**DOI:** 10.1371/journal.pone.0015587

**Published:** 2010-12-28

**Authors:** Asfia Qureshi, Marimuthu Subathra, Angus Grey, Kevin Schey, Maurizio Del Poeta, Chiara Luberto

**Affiliations:** 1 Department of Biochemistry and Molecular Biology, Medical University of South Carolina, Charleston, South Carolina, United States of America; 2 Mass Spectrometry Research Center, Department of Biochemistry, Vanderbilt University, Nashville, Tennessee, United States of America; 3 Departments of Microbiology and Immunology, Medical University of South Carolina, Charleston, South Carolina, United States of America; 4 Division of Infectious Diseases, Medical University of South Carolina, Charleston, South Carolina, United States of America; Research Institute for Children and the Louisiana State University Health Sciences Center, United States of America

## Abstract

The key host cellular pathway(s) necessary to control the infection caused by inhalation of the environmental fungal pathogen *Cryptococcus neoformans* are still largely unknown. Here we have identified that the sphingolipid pathway in neutrophils is required for them to exert their killing activity on the fungus. In particular, using both pharmacological and genetic approaches, we show that inhibition of sphingomyelin synthase (SMS) activity profoundly impairs the killing ability of neutrophils by preventing the extracellular release of an antifungal factor(s). We next found that inhibition of protein kinase D (PKD), which controls vesicular sorting and secretion and is regulated by diacylglycerol (DAG) produced by SMS, totally blocks the extracellular killing activity of neutrophils against *C. neoformans*. The expression of *SMS* genes, SMS activity and the levels of the lipids regulated by SMS (namely sphingomyelin (SM) and DAG) are up-regulated during neutrophil differentiation. Finally, tissue imaging of lungs infected with *C. neoformans* using matrix-assisted laser desorption-ionization mass spectrometry (MALDI-MS), revealed that specific SM species are associated with neutrophil infiltration at the site of the infection. This study establishes a key role for SMS in the regulation of the killing activity of neutrophils against *C. neoformans* through a DAG-PKD dependent mechanism, and provides, for the first time, new insights into the protective role of host sphingolipids against a fungal infection.

## Introduction

One hundred and sixteen years after the discovery of *Cryptococcus neoformans* from fermented peach juice by the Italian Sanfelice [1], and shortly thereafter from the tibial lesions of a German patient by Busse [2] and Buschke [3], the total containment of a cryptococcal infection by the host remains elusive. *C. neoformans* is an environmental fungus found worldwide that commonly strikes those individuals having compromised immune systems [4], although immunocompetent subjects can also be affected [5]. Infection is initiated upon inhalation of spores or desiccated fungi, and in the lung *C. neoformans* proliferates in the alveolar space. Whereas in immunocompetent subjects the infection is, for the most part, contained in the lung, in immunocompromised subjects dissemination of fungal cells from the lung to the brain leads to the development of a life-threatening meningoencephalitis [6], [7], [8]. Indeed, deaths by cryptococcosis among HIV-infected patients in sub-Saharan Africa are more frequent than deaths by tuberculosis [9].

Almost all reviews on host defense against *C. neoformans* emphasize the role of cell-mediated immunity (CMI), which is critical for containment of fungal cells through the activation of macrophages and neutrophils resulting in granuloma formation [10], [11], [12]. Although several studies have elucidated the role and mechanisms by which macrophages, and especially alveolar macrophages, control *C. neoformans* infection [6], [13], [14], [15], [16], [17], [18], [19], [20], very little is known on the mechanisms by which neutrophils neutralize *C. neoformans*. Neutrophils are more effective killers than macrophages against fungi [21], [22] and they possess the innate ability to kill microbes without the activation of a cellular mediated immune response. Very interestingly, transfusion of human neutrophils, as differentiated HL-60 cells, significantly improves survival of mice challenged with fungal organisms [23], [24], suggesting that these cells are able to control the infection through their antifungal activity. Although neutrophils efficiently kill fungi, the host signaling mechanism(s) required for this killing activity is unknown. The elucidation of these mechanisms regulating their antifungal properties would not only improve our understanding of the host-pathogen interaction but it could facilitate the use of such immune cells in a clinical setting.

In recent years, sphingolipids, and sphingolipid-metabolizing enzymes, have emerged as key regulators of many physiological and pathological cellular processes. The study of the role of sphingolipids in the regulation of infectious diseases is also a rapidly emerging area of research, but, until very recently, most of these studies have focused on the role of microbial sphingolipids in the ability of the microbe to cause infection [25], [26]. Very few studies have addressed whether host sphingolipids are also involved in the regulation of microbial pathogenesis and nothing is known about the role of host sphingolipids on fungal infections.

An intriguing sphingolipid metabolizing activity is carried out by sphingomyelin synthase (SMS), which is encoded by two genes: *SMS1* and *SMS2* [27], [28], [29]. SMS transfers a choline phosphate moiety from phosphatidylcholine (PC) to ceramide, thereby producing sphingomyelin (SM) and diacylglycerol (DAG) [17], [29], [30]. This class of enzymes is particularly important because not only does it produce SM, a key component of cellular membranes, but also because it regulates the level of two bioactive lipid molecules, such as ceramide and DAG. Since (a) ceramide can regulate transcription factors, such as NF-κB involved in cytokine production [31], (b) DAG controls antifungal activity by neutrophils through reactive oxygen species (ROS) production [32], and (c) SM has been implicated in controlling the host immune response in phagocytic cells [33], in this paper we studied the role of SMS in neutrophils against *C. neoformans*.

We show herein that inhibition of SMS activity profoundly impairs the ability of neutrophils to kill *C. neoformans* cells. We also provide evidence supporting a mechanism by which SMS regulates neutrophil killing against *C. neoformans* through the production of DAG and the consequent activation of protein kinase D (PKD).

## Materials and Methods

### Ethics Statement

This study was carried out in strict accordance with the recommendations in the Guide for the Care and Use of Laboratory Animals of the National Institutes of Health. The protocol was approved by the Medical University of South Carolina Institutional Animal Care and Use Committee (Permit Number: 2019). All animal procedures were performed according to the approved protocol, and all efforts were made to minimize suffering.

### Materials, strains and growing media

*Cryptococcus neoformans* variety *grubii* serotype A strain H99 (WT) was used in this study, and routinely grown in yeast extract/peptone/2% dextrose-rich (YPD-rich) medium. HL-60 cells (ATCC® CCL-240™) were cultured at 37°C, 5% CO_2_ in RPMI 1640, supplemented with L-glutamine, 20% heat-inactivated FBS, and 1% penicillin and streptomycin. RPMI 1640 medium, FBS and penicillin-streptomycin were from Gibco/Invitrogen; pooled human serum, retinoic acid and DMSO were from Sigma. D609 was purchased from Enzo Life Sciences. CID755673 was purchased from Tocris Bioscience; 1,2-Dioctanoyl-*sn*-glycerol (DiC8) was from Cayman Chemical. NBD [N-(7-nitrobenz-2-oxa-1,3-diazol-4-yl)]-C_6_-ceramide was purchased from Molecular Probes. Phosphatidylcholine (PC) was purchased from Avanti Polar Lipids.

### HL-60 killing assay

To quantify the effect of HL-60 cells on *C. neoformans* (H99), the killing assay described by Spellberg *et al* [24] was used with slight modification. HL-60 cells were differentiated by incubation in the presence of 1.3% (v/v) DMSO and 2.5 µM retinoic acid up to 72 h in growth medium. Cells were then washed with RPMI serum free medium and resuspended in fresh RPMI containing 10% pooled human serum. Then, 8×10^4^ phagocytes were co-cultured with 4×10^3^ *C. neoformans* in a total volume of 1 ml (20∶1 ratio HL-60:*C. neoformans*) for 6 h at 37°C. At the end of the incubation, the cultures were sonicated using a sonic dismembrator Model 500 (Fisher Scientific) at 10% amplitude twice (15 seconds each) on ice, serially diluted and streaked onto YPD agar, and incubated for 48 h at 30°C. CFUs were counted to assess killing of *C. neoformans* compared with control cultures of *C. neoformans* alone with no HL-60 cells.

### SMS inhibition assay

To test whether SMS activity is required for activated HL-60 cells to kill *C. neoformans*, the pharmacological SMS inhibitor D609 was used. Following incubation with 1.3% (v/v) DMSO and 2.5 µM retinoic acid for 72 h, cells were washed, and 8×10^4^ phagocytes in 1 ml were resuspended in fresh RPMI and 10% pooled human serum and incubated with D609 (50 µg/ml) for 2 h. Then, 4×10^3^ *C. neoformans* cells were added (20∶1 ratio HL-60:*C. neoformans*) for an additional 4 h. At the end of the incubation, the cultures were sonicated, serially diluted and streaked onto YPD agar, and incubated for 48 h at 30°C. CFUs were counted to assess killing of *C. neoformans* compared with control cultures of *C. neoformans* alone with no HL-60 cells.

### SMS activity assay

HL-60 cells (undifferentiated and differentiated for 72 h) were collected and the pellet rinsed with PBS then resuspended in 100 µl of ice-cold lysis buffer. For the experiments in which D609 (50 µg/ml) was used, the inhibitor was incubated with HL-60 cells for 6 h post-differentiation, at 37°C prior to cell collection. The lysis buffer consisted of 25 mM Tris/HCl (pH 7.4), 5 mM EDTA, 1% phosphatase inhibitor and 1% protease inhibitor (both from Thermo Scientific). Cell lysates were sonicated using a sonic dismembrator Model 500 (Fisher Scientific) at 10% amplitude twice (15 seconds each) on ice, then centrifuged at 400 *g* for 5 minutes at 4°C. The supernatant was used for measuring enzymatic activity. Protein concentrations were determined using the Bio-Rad assay. The SMS assay was performed using 150 µg of protein. The substrate was prepared as a mixture of 40 µM NBD-C6 ceramide and 200 µM phosphatidylcholine (PC) resuspended in 100 mM Tris/HCl (pH 7.4), 50 mM KCl and 1 mM EDTA by sonication (ultrasonic water bath) and vortexing in turn until clear. The substrate was diluted 1∶1 with the proteins resuspended in lysis buffer (final incubation volume of 200 µl), and the incubation was carried out for 30 min at 30°C. The reaction was stopped on ice by addition of 3 vol. of chloroform/methanol (1∶1 v/v). After vortexing, the phases were clarified by centrifugation at 2400 *g* for 5 min. The lower phase was transferred to new tubes, dried down, and the lipids resuspended with 40 µl of chloroform/methanol (2∶1 v/v) and separated by TLC in chloroform:/methanol/15 mM CaCl_2_ (90∶52.5∶12 by vol.). Fluorescence was measured using a Storm 860 Imaging Analysis System from Amersham Biosciences (U.K.). Results were analyzed using ImageQuant software from Amersham Biosciences.

### Down-regulation of *SMS1* and *SMS2*

Down-regulation of *SMS1* and *SMS2* was achieved with siRNA oligonucleotides targeting *SMS1*: ACAGCTTACCTAGATCATAAA (*SMS1.2* siRNA) and CACACTATGGCCAATCAGCAA (*SMS1.4* siRNA), or *SMS2*: AAGGCACCAAAAAGTACCCGG (*SMS2.0* siRNA), and ACCGTCATGATCACAGTTGTA (*SMS2.3* siRNA) synthesized by Qiagen, and by using Nucleofector Solution V nucleofection reagent (Lonza). The non-specific All Star siRNA sequence (SCR; scrambled siRNA) was used as control (Qiagen). Typically, 2×10^6^ HL-60 cells from an exponentially growing culture not exceeding 25 passages were nucleofected with 4.5 µg siRNA according to the manufacturer's directions with the following modification. After 30 minutes of incubation at 37°C, 5% CO_2_ in 500 µl of growth medium, cells were seeded at 1×10^5^ cells/ml. Cells were then treated with 1.3% (v/v) DMSO and 2.5 µM retinoic acid. After 48 h, cells were collected and frozen at −80°C for RT-PCR. For the killing assay, cells were spun at 800 *g* for 5 minutes at RT. Pellets were resuspended with RPMI containing 10% human serum. Eight ×10^4^ differentiated cells were incubated with *C. neoformans* for an additional 4 h in a ratio of 20∶1 HL-60:*C. neoformans* in a total volume of 1 ml. At the end of the incubation, the cultures were serially diluted and streaked onto YPD agar, and incubated for 48 h at 30°C. CFUs were counted to assess killing of *C. neoformans* compared with control cultures of *C. neoformans* alone with no HL-60 cells.

### Real-time RT-PCR

Total RNA was extracted using an RNeasy Mini Kit (Qiagen) following the manufacturer's suggested protocol (including the optional DNase and wash steps). Real time PCR was performed as indicated by Villani *et al* [30]. The results were normalized to an internal control gene, glyceraldehyde-3 phosphate dehydrogenase (GAPDH). The real-time RT–PCR results were analysed using Q-Gene® software, which expresses data as the means of normalized expression. The primers were designed using PerkinElmer Primer Express® software. Primer sequences were as follows: GAPDH, forward: 5′-CACCAGGGCTGCTTTTAACTCTGGTA-3′, reverse: 5′-CCTTGACGGTGCCATGGAATTTGC-3′; SMS1, forward: 5′-GCCAGGACTTGATCAACCTAACC-3′, reverse: 5′-CCATTGGCATGGCCGTTCTTG-3′; SMS2, forward: 5′-TC-ACCCAGTGGCTGTTTCTGA-3′, reverse: 5′-TGCATTCCAGGCACAGGTAGA-3′.

### Flow cytometry analysis

HL-60 cells (1×10^5^ cells/ml, total 20 ml) were treated with 1.3% (v/v) DMSO and 2.5 µM retinoic acid. At the indicated time points cells were collected and analyzed for the CD11b surface marker which is constitutively expressed in neutrophils by flow cytometry using a BD FACSCalibur™ platform with fluorescein isothiocyanate (FITC) labeled antibodies (AbD Serotec) according to the manufacturer's recommended dilution and protocol. Briefly, 1×10^6^ cells (in 100 µl) were incubated with the 500 ng of antibody in PBS containing 2% FBS for 20 minutes on ice, washed with PBS supplemented with 2% FBS, and re-suspended with 500 µl of PBS supplemented with 2% FBS and analyzed by flow cytometry. The concentration of the antibodies was optimized using positive controls, and negative isotype controls were employed to exclude non-specific binding. For experiments using siRNA, differentiation was initiated immediately after the siRNA transfection and allowed to proceed for 48 hours.

### HL-60 medium killing assay

Undifferentiated and differentiated HL-60 cells were washed with RPMI serum free medium and re-suspended in fresh RPMI supplemented with 10% pooled human serum. Next, 8×10^4^ HL-60 cells were incubated in 1 ml for 6 hours in the presence or absence of inhibitors (D609 or PKD1 inhibitor CID755673). Conditioned media (900 µl) were then collected by centrifugation and 4×10^3^ *C. neoformans* cells/ml added and allowed to incubate for 4 h at 37°C. At the end of the incubation, the medium was serially diluted and streaked onto YPD agar, and incubated for 48 h at 30°C. CFUs were counted to assess killing of *C. neoformans* in the absence or presence of inhibitors compared with control cultures of *C. neoformans* alone in fresh medium.

### Down-regulation of *PKD1* and *PKD2*

Down-regulation of *PKD1* and *PKD2* was achieved with siRNA oligonucleotides targeting *PKD1* (5′GUCGAGAGAAGAGGUCAAATT3′), or *PKD2* (5′GCAAAGACUGCAAGUUUAATT3′, *PKD2.1;* 5′GGACAUCAAUGACCAGAUCTT-3′, *PKD2.2*) synthesized by Qiagen. The non-specific All Star siRNA sequence (SCR; scrambled siRNA) was used as control (Qiagen). Down-regulation of *PKD1* and *PKD2* was achieved by nucleofecting cells with 4.5 µg siRNA as described for SMS. Cells were then treated with 1.3% (v/v) DMSO and 2.5 µM retinoic acid. After 48 h, cells were collected and frozen at −80°C for Western blotting, or used immediately for incubation with *C. neoformans* in the killing assay as previously described for SMS down-regulation.

### Western Blot of PKD

HL-60 cells (1×10^5^ cells/ml, total 40 ml) were treated with 1.3% (v/v) DMSO and 2.5 µM retinoic acid. At the indicated time points (24, 48 and 72 hrs), 6×10^6^ cells were collected and PKD1, PKD2 and PKD3 protein expression was assayed by Western blotting. For experiments with siRNA, cells were nucleofected with SCR, PKD1, PKD2.1 and PKD2.2 siRNA as described for SMS. After 48 hours of differentiation cells were collected and processed for western blotting. Three million cells were harvested on ice, washed in PBS and resuspended in 80 µl of doubly distilled water containing Pierce Halt protease inhibitor and 5 mM PMSF. Cells were then lysed by sonication using a sonic dismembrator (conditions described above; optimization experiment showed 100% cell lysis with these conditions). Protein concentration was determined using the Bio-Rad protein determination assay reagent. Fifty µg of total proteins for PKD1 and 10 µg of total proteins for PKD2 and PKD3 were separated by 10% SDS-PAGE under reducing conditions and transferred to nitrocellulose membranes. Membranes were blocked with 2% BSA containing PBS-Tween 0.1% for 1 hour at room temperature. Membranes were then incubated with PKD1, PKD2, PKD3 antibodies (1∶1000 dilution) (PKD1 from Cell Signaling Technology, PKD2 & PKD3 from Bethyl Laboratories) overnight with continuous shaking at 4°C. After extensive washings, membranes were next incubated with peroxidase-conjugated donkey-anti-rabbit (1∶10,000, Santa Cruz Biotechnology Inc.) in 2% BSA PBS-Tween 0.1% for 1 hour at room temperature. Signals were visualized using SuperSignal West Femto (Thermo Scientific) and exposure to Kodak BioMax MR Film. GAPDH was used as a loading control. The intensity of the signal was determined by densitometry using Labworks Image Acquisition and Analysis software from UVP BioImaging Systems, version 4.5.

### HL-60 killing assay in the presence of DiC8

To test whether exposure of HL-60 cells to the DAG analogue 1,2-Dioctanoyl-*sn*-glycerol (DiC8) improves the killing effect observed by differentiated HL-60 cells, following incubation with 1.3% (v/v) DMSO and 2.5 µM retinoic acid as described above, 8×10^4^ cells were washed, re-suspended in 1 ml fresh RPMI containing 10% pooled human serum and incubated with varying concentrations of DiC8 for 2 h. Then, 4×10^3^ *C. neoformans* cells were added (20∶1 ratio HL-60:*C. neoformans*) for an additional 4 h. At the end of the incubation, the cultures were serially diluted and streaked onto YPD agar, and incubated for 48 h at 30°C. CFUs were counted to assess killing of *C. neoformans* compared with control cultures of *C. neoformans* alone with no HL-60 cells.

### SM, ceramide and DAG determination

Undifferentiated and differentiated HL-60 cells (1×10^6^) were washed with PBS, and frozen pellets were submitted for mass spectral data acquisition at the Medical University of South Carolina Lipidomics Facility. For lipid analysis in lung tissues, lungs from 2 mice from each time point were homogenized in 5 ml homogenization buffer consisting of 0.25 M sucrose, 0.5 mM EDTA, 25 mM KCl, and 50 mM TRIS-HCl at pH 7.4. Then, 1 mg of homogenate was used for the mass spectrometry analysis whereas an aliquot was used for the quantitation of GAPDH by Western blot using the LabWorks Image Acquisition and Analysis software from UVP BioImaging Systems, version 4.5. Lipids were extracted and analyzed using established protocols in the facility [34], [35].

### Animal studies

Four- to six-week old CBA/J mice from Jackson Laboratories were used for this study. Mice were anesthesized by intraperitoneal injection of 60 µl of a xylazine-ketamine mixture containing 5 mg xylazine and 95 mg ketamine per kg of body weight. The wild-type strain (H99) of *C. neoformans* was grown in YPD medium for 24 h at 30°C. The fungal cells were harvested, washed three times in PBS, and resuspended in PBS at a concentration of 2.5×10^7^ cells/ml. Mice were infected intranasally with 20 µl containing 5×10^5^ cells. Mice were fed *ad libitum* and monitored by inspection twice a day. Mice that appeared moribund or in pain were sacrificed using CO_2_ inhalation followed by cervical dislocation. All animal procedures were approved by the Medical University of South Carolina Institutional Animal Care and Use Committee and followed the guidelines of the American Veterinary Medical Association.

### Tissue sectioning and sample preparation

At 6, 12 and 18 days post infection, mice were euthanized and lungs were harvested and flash-frozen in dry-ice/ethanol, then stored at -80°C until ready for use. The organ was attached to the cryostat sample stage using a small bead of optimal cutting temperature compound (OCT) at the base of the tissue only. The tissue was sectioned to a thickness of 20 µm at a temperature of −26°C using a cryotome (Microm HM 550, Walldorf, Germany). The sections were thaw-mounted onto conductive indium tin oxide (ITO) coated conductive glass slides for mass spectral analysis (Bruker Daltonics, Billerica, MA), or superfrosted glass slides for mucicarmine and hematoxylin and eosin staining. The resistance of the ITO coated microscope slide was 40 Ω over a distance of 1 cm. The tissue was allowed to warm on the microscope slides for 10 s before refreezing and storage at −80°C. For mass spectrometric analysis, the tissue sections were removed from the freezer and placed in a dessicator for 30 min prior to matrix deposition. A freshly prepared solution of 2,5-dihydroxybenzoic acid (DHB) (Sigma), (40 mg/ml in 70% ethanol), was applied to the tissue by repeated cycles using a TLC sprayer. Each spray cycle was followed by 45–60 s of drying time, and the cycle repeated until an even coverage of matrix across the entire tissue was achieved.

### MALDI Mass Spectrometry SM Imaging

MALDI mass spectral analysis was carried out using a reflector time-of-flight mass spectrometer (Bruker Autoflex III TOF-TOF, Bruker Daltonik, Bremen, Germany) operating in positive ion mode with a +20 kV accelerating potential. The laser beam size was set to medium, and operated at 200 Hz. Using Bruker Peptide Standard 1 (Bruker Daltonik, Bremen, Germany), a linear, external calibration was applied to the instrument before data collection. Mass spectral data sets were acquired over each whole mouse lung using flexImaging™ software (Bruker Daltonik, Bremen, Germany) in the mass range of *m/z* 500–1200 with a raster step size of 100 µm and 250 laser shots per spectrum. After data acquisition, molecular images were reconstituted using flexImaging™ software. Each data set consists of approximately 4,000 individual sampling locations, each representing one pixel in the resultant image. Data was normalized using flexImaging™ software, and each *m/z* signal plotted ±0.5 mass units. For display purposes, signals between sampling locations were interpolated and pixel intensities were scaled to utilize the entire dynamic range. Tandem mass spectrometry was used to identify signals detected in the MALDI imaging data sets. Lipids to be identified were extracted from mouse lung tissue by homogenization of the tissue in 70% ethanol. Samples were centrifuged at 45,000 rpm for 30 minutes at 4°C using a Beckman Optima TL Ultracentrifuge with a TLA45 rotor (Beckman Coulter, Inc., Fullerton, CA), and the supernatant containing extracted lipids removed. Samples were concentrated using a speed-vac (Labconco, Kansas City, MO), and spotted on a MALDI plate using 40 mg/ml DHB in 70% ethanol. Standard solutions of known lipids were also spotted in a similar manner. A timed ion gate was used for precursor ion selection and the fragments generated were further accelerated with 19 kV in the LIFT cell, and detected following passage through the reflectron. No CID gas was used for fragmentation of the precursor ions. Signals in the MALDI tissue imaging data set were identified based on matching fragmentation spectra of lipids extracted from the tissue and prepared lipid standard solutions.

### Neutrophil killing assay

Approximately 6.4×10^4^ fresh human normal peripheral blood-neutrophils per ml (AllCells) were incubated in 124 µl sterile filtered PBS supplemented with 10% pooled human serum for 2 hours in the absence or presence of inhibitors (SMS inhibitor D609 or PKD1 inhibitor CID755673). Then, 10 µl of 4×10^4^ *C. neoformans* cells/ml were added (20∶1 ratio neutrophils:*C. neoformans*) for an additional 4 h. At the end of the incubation, the cultures were sonicated, serially diluted and streaked onto YPD agar, and incubated for 48 h at 30°C. CFUs were counted to assess killing of *C. neoformans* compared with control cultures of *C. neoformans* alone with no neutrophils. Murine neutrophils were obtained from CBA/J mice by Ficoll-Hypaque separation according to the procedure of Culpitt [36]. Mouse sera was obtained from CBA/J mice. The killing assay for murine neutrophils was identical to that described for human neutrophils.

### Statistics

All experiments were performed at least in triplicate. Statistical analysis of the data were performed using Student's *t*-test, and *P*<0.05 was considered statistically significant.

## Results

### Differentiated HL-60 cells kill *C. neoformans*

**Figure 1:**
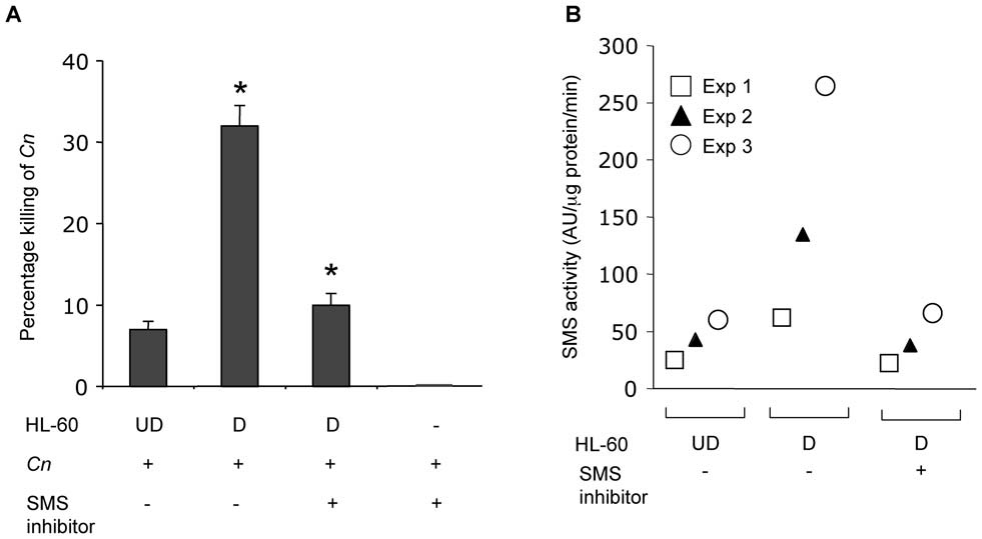
(**A**). Killing activity of HL-60 cells against *C. neoformans* (*Cn*) increases with differentiation and it is abrogated by inhibition of sphingomyelin synthase activity. Percentage killing of *C. neoformans* wild-type H99 by HL-60 cells undifferentiated (UD) and differentiated (D) with retinoic acid and DMSO. *, *P*<0.05. Treatment with sphingomyelin synthase inhibitor D609 completely abolished the killing activity of differentiated (D) cells. D609 has no effect on *Cn* cells. (**B**) Differentiation of HL-60 cells increases SMS activity. Sphingomyelin synthase (SMS) activity increases in HL-60 differentiated (D) compared to undifferentiated (UD) cells. Treatment with SMS inhibitor D609 decreases SMS activity of HL-60 D. Results show are from 3 independent experiments.

To study how neutrophils control *C. neoformans*, we used an *in vitro* human cell system that was characterized a few years ago in an elegant study by Spellberg and co-workers [24]. Therefore, to determine the anticryptococcal activity of neutrophils, 8×10^4^ HL-60 phagocytes (differentiated or undifferentiated) were co-cultured with 4×10^3^ *C. neoformans* wild-type H99 (20∶1 ratio) as described in the Materials and Methods Section. Fungal cells were serially passaged twice in yeast extract/peptone/dextrose (YPD) broth and washed twice with phosphate buffered saline (PBS) prior to the co-incubation. Following co-incubation, the culture was sonicated, serially diluted and plated onto YPD agar and incubated at 30°C for 48 hours to determine colony forming unit (CFU) counts in order to assess *C. neoformans* viability. Sonication conditions were optimized to achieve lysis of mammalian cells and 100% viability of fungal cells (data not shown). Results are expressed as the percent reduction of CFU from fungal-phagocyte co-cultures *versus* simultaneous culture of *C. neoformans* without phagocytes. Experiments were performed in triplicate and repeated three times. Since *C. neoformans* alone in this minimum medium does not replicate during the period of incubation, a reduction of *C. neoformans* cell number in the presence of differentiated HL-60 indicate killing of *C. neoformans* (percentage killing). A ∼4 fold increase of killing activity against *C. neoformans* was observed when HL-60 were differentiated (32%) as compared to undifferentiated (7%) (**Figure 1A**). These results suggest that, in addition to *Candida albicans* (*Ca)*, differentiated HL-60 can also efficiently kill *C. neoformans*. Interestingly, the killing activity of differentiated HL-60 against *Ca* increased by only ∼2 fold compared to the one observed with undifferentiated cells [24]. This suggests that the killing activity of differentiated HL-60 towards *C. neoformans* seems to be more pronounced than the one observed against *Ca*.

### Inhibition of SMS activity totally abrogated the killing activity by HL-60 differentiated cells

Since SM, ceramide and DAG are important lipids involved in controlling the host immune responses [31], [37], [38], [39], [40], [41], [42], we investigated whether SMS activity is required for neutrophils to kill *C. neoformans*. To address this question, SMS activity was inhibited with the pharmacological SMS inhibitor D609 [34], [43], [44] and *C. neoformans* survival examined in treated *versus* untreated cells. After 72 hours of differentiation, 8×10^4^ phagocytes were incubated with 50 µg/ml of D609 for 2 hours (dose and time adequate to inhibit SMS activity in various cell lines) [44], [45]. Next, 4×10^3^ *C. neoformans* wild-type H99 cells were added for an additional 4 hours at 37°C in RPMI +10% pooled human serum. During this time, D609 was still present in the medium in order to maintain SMS activity as low as possible even in the presence of *C. neoformans* cells. As control, the effect of D609 (50 µg/ml) was determined on viability of either *C. neoformans* cells alone (CFU after 4 hours incubation) or HL-60 cells in the absence of *C. neoformans* (trypan blue exclusion after 6 hours of incubation). In both cases, no cytotoxic effect was induced by D609 (**Figure 1A**). As shown in **Figure 1A**, D609 totally inhibited the killing activity of differentiated HL-60 cells, suggesting that differentiated HL-60 cells kill *C. neoformans* through an SMS-mediated mechanism.

### SMS activity increases in differentiated compared to undifferentiated HL-60 cells

To study whether SMS could be involved in the killing activity of HL-60, it was examined whether differentiation of HL-60 would increase SMS activity. Following differentiation of HL-60, proteins were extracted and assayed for SMS activity using well-established and published methods [30]. SMS activity was found to be up-regulated in differentiated compared to undifferentiated HL-60 and incubation with the SMS inhibitor D609 inhibited SMS activity of differentiated HL-60 cells (**Figure 1B**).

### Down-regulation of either SMS1 or SMS2 decreases killing of C. neoformans by differentiated HL-60 cells

**Figure 2:**
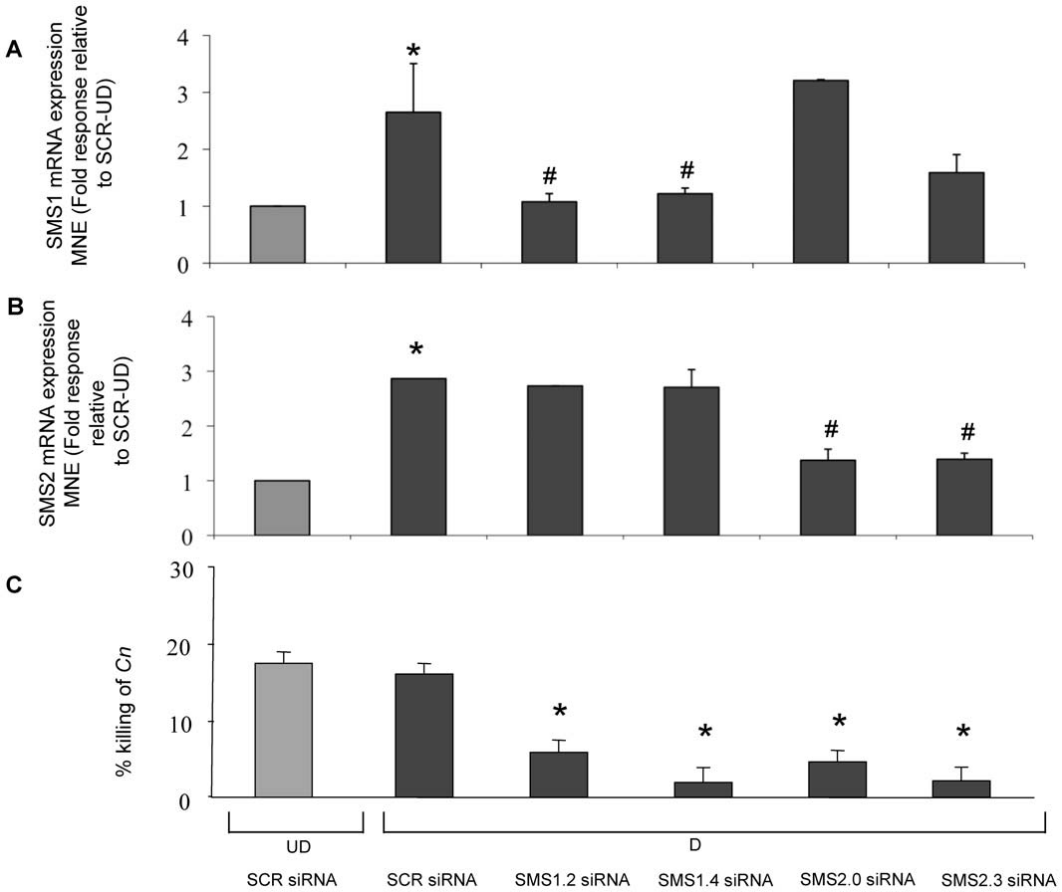
Effect of modulation of either *SMS1* or *SMS2* during differentiation of HL-60 cells. Two million HL-60 cells were transfected with 4.5 µg of SCR, SMS1 siRNA (1.2 and 1.4), and SMS2 siRNA (2.0, 2.3) by nucleofection. Differentiation and down-regulation were induced for 48 hours. (**A** and **B**) Total RNA was extracted, and RT-PCR was performed using specific primers for *SMS1* or *SMS2* and *GAPDH*. The RT-PCR results were analyzed using Q-gene software which expresses data as the means of normalized expression (Fold response relative to SCR-UD). *SMS1* mRNA expression was down-regulated by both *SMS1.2* and *SMS1.4* siRNA (A) and *SMS2* mRNA expression was down-regulated by both *SMS2.0* and *SMS 2.3* siRNA (B). Data are the results of at least 3 independent experiments; error bars represent SD and * *P*<0.05 compared to SCR siRNA undifferentiated cells; # *P*<0.05 compared with SCR siRNA differentiated cells. (**C**) Inhibition of either *SMS1* or *SMS2* mRNA by siRNA blocks the killing activity of differentiated HL-60 cells; results are representative of at least 3 independent experiments. UD: undifferentiated cells; D: differentiated cells; SCR: scrambled control siRNA. MNE: mean of normalized expression. * *P*<0.05 compared with SCR siRNA differentiated cells.

In mammalian cells SMS activity is encoded by two genes: *SMS1* and *SMS2* [27], and in differentiated HL-60 cells both genes are expressed (**Figure S1**). Interestingly and in agreement with the increase of total SMS activity observed in differentiated HL-60 cells (**Figure 1B**), both *SMS1* and *SMS2* expression increased in the course of the differentiation process (**Figure S1**). In order to study the role of these genes in the antifungal activity of differentiated HL-60, short interfering RNA (siRNA) was used. The siRNA treatment was optimized so that, after down-regulation, *SMSs* expression in differentiated cells would approximate the levels found in undifferentiated cells. Cells were treated with siRNA against *SMS1 (SMS 1.2 and SMS 1.4)*, *SMS2 (SMS2.0 and SMS 2.3)* or scrambled control (*SCR*) together with the agents required to induce differentiation towards the granulocytic lineage. The Lonza® Nucleofector® optimized protocol for HL-60 cells was used to transfer the siRNA into the cells. After optimization experiments, it was found that transfection of 4.5 µg of siRNA for 48 hours was the optimal concentration to down-regulate gene levels to those found in undifferentiated cells (**Figure 2A and B**). Importantly, no significant changes in *SMS2* levels were observed when *SMS1* was down-regulated (**Figure 2A** **)** and vice versa (**Figure 2B** **)** indicating the absence of cross-target effects.

Thus, to test the effect of down-regulation of *SMS1* or *SMS2* on the extracellular killing of *C. neoformans* by differentiated HL-60, SMS down-regulated cells (48 h of siRNA transfection and differentiation) were incubated with *C. neoformans* for 4 h in a 20∶1 ratio (HL-60:*C. neoformans*), as previously described. The cultures were then collected, processed and plated for fungal CFUs. As a control for fungal growth, *C. neoformans* was incubated in the absence of HL-60 cells. The killing activity is represented by the percentage of *C. neoformans* CFU recovered from *C. neoformans* incubated alone minus the *C. neoformans* CFU recovered from *C. neoformans* cells incubated with differentiated HL-60 from each experimental group, divided by *C. neoformans* CFU recovered from *C. neoformans* incubated alone (**Figure 2C**). It was found that both *SMS1* (*SMS1.3* and *1.4*) and *SMS2* (*SMS2.0* and *2.3*) siRNA significantly inhibited the killing of *C. neoformans* cells. These results provide further evidence that differentiated HL-60 cells kill *C. neoformans* by an SMS-mediated mechanism.

### SMS knockdown by siRNA does not affect HL-60 cell differentiation

**Figure 3:**
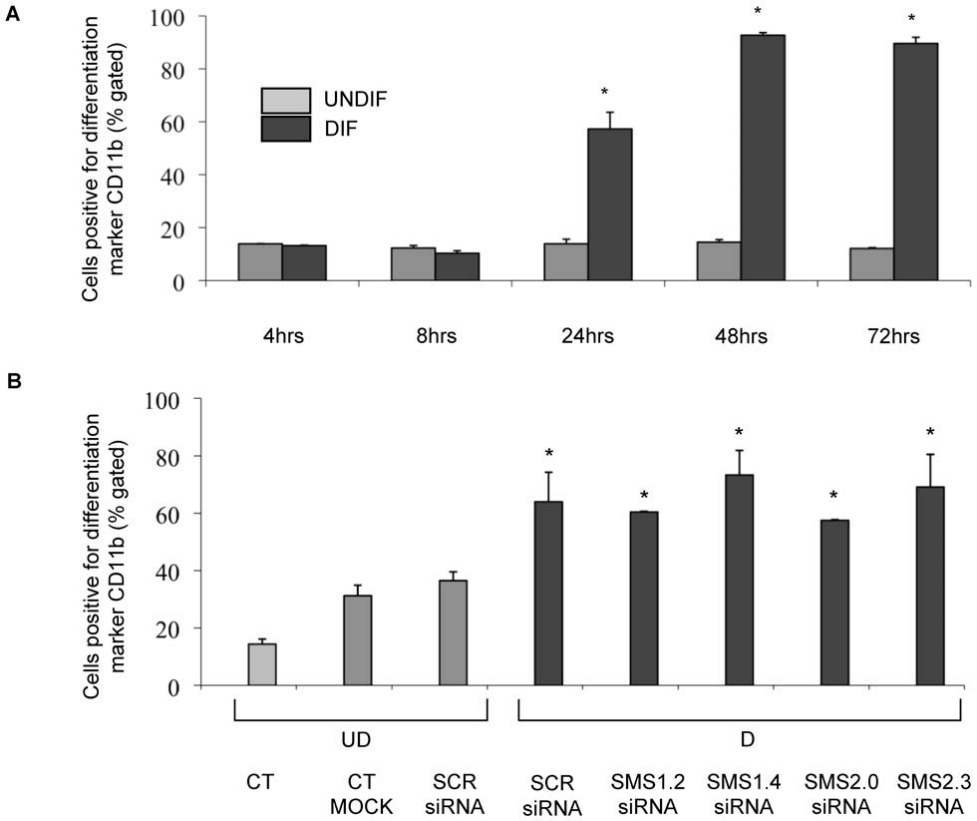
Analysis of CD11b differentiation marker by flow cytometry. (**A**) HL-60 cells were plated at 1×10^5^ cells/mL and differentiation induced. Undifferentiated cells received vehicle solution for the retinoic acid. Differentiated and undifferentiated cells were collected at the indicated time points and processed for flow cytometric analysis of CD11b positive cells. * *P*<0.05 compared with undifferentiated time-matched samples. (**B**) Effect of modulation of either *SMS1* or *SMS2* on differentiation of HL-60 cells by analysis of CD11b differentiation marker using flow cytometry. Two million HL-60 cells were transfected with 4.5 µg of SCR siRNA, *SMS1* siRNA (1.2 and 1.4), and *SMS2* siRNA (2.0, 2.3) by nucleofection. Downregulation and differentiation proceeded for 48 hours. Cells were then collected and processed for flow cytometric analysis of CD11b. * *P*<0.05 compared with SCR siRNA undifferentiated cells. UD: undifferentiated cells; D: differentiated cells; CT MOCK: control for transfection reagent; SCR: control scrambled siRNA.

In order to demonstrate that knockdown of *SMS1* and *SMS2* by siRNA did not affect differentiation, expression of the neutrophil cell-surface marker CD11b was determined. First, expression of CD11b was confirmed during the neutrophilic differentiation of HL-60 cells (**Figure 3A**). Indeed, expression of CD11b reached a maximum after 48 h and this maximum was sustained at 72 h. Therefore, following transfection of SCR, SMS1 or SMS2 siRNA, the cells were tested for CD11b expression after 48 hours of differentiation (**Figure 3B**). Of note, transfection of HL-60 cells by itself had the tendency to elevate CD11b expression, albeit not significantly. Nevertheless, a significant increase of CD11b expression was observed after differentiation of scrambled siRNA treated cells (UD SCR siRNA versus D SCR siRNA). Most importantly, expression of the CD11b marker upon differentiation was not affected by *SMS1* or *SMS2* siRNA. These results suggest that the observed increase in SMS activity during differentiation (**Figure 1B**) is rather a consequence and not an inducer of neutrophilic differentiation of HL-60 cells and that the effect observed after down-regulation of *SMS1* or *SMS2* on the killing activity of HL-60 cells (**Figure 2C**) was not due to an impairment of the differentiation process.

### The killing activity of differentiated HL-60 is due to antifungal factors present in the medium

To decipher the mechanism by which differentiated HL-60 cells kill *C. neoformans*, it was examined whether these phagocytes would internalize *C. neoformans* during the time of incubation and, thus, kill them intracellularly through the phagolysosome, or whether the differentiation process would promote the release of some host factor(s) in the medium that would kill *C. neoformans* extracellularly. To address whether phagocytosis would occur, 8×10^4^ differentiated HL-60 cells were incubated with 4×10^3^ *C. neoformans* cells, as described above for the killing experiment. At different time points (1, 2, 3 and 4 hours of co-incubation) the phagocytic index was determined by microscopic observation. At all time points, not a single *C. neoformans* cell was observed within HL-60 cells (differentiated or undifferentiated) (data not shown). This is most likely due to the unfavorable ratio of *C. neoformans* versus HL-60 cells (1∶20) used in the assay. These results suggest that the killing activity of HL-60 under these *in vitro* conditions is not mediated by phagocytosis and intracellular killing of *C. neoformans*.

**Figure 4:**
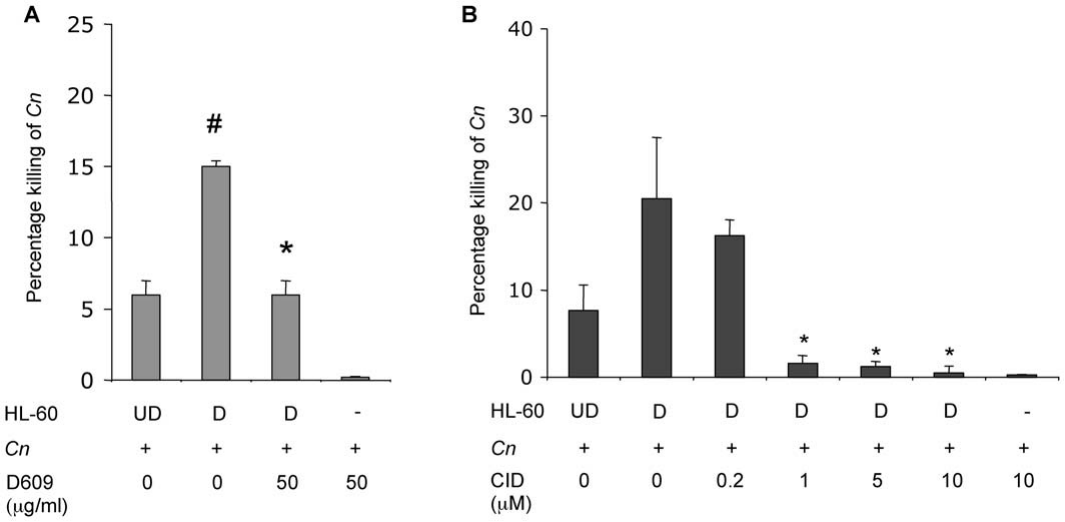
Effect of conditioned media on the killing activity of HL-60 cells. (**A**) Percentage killing of *C. neoformans* wild-type H99 (*Cn*) by conditioned media collected from HL-60 cells undifferentiated (UD), differentiated (D), from HL60 D treated with D609 or by D609 itself. (**B**) Effect of PKD inhibitor on the killing activity of differentiated HL-60 cells against *C. neoformans* (*Cn*) or against *C. neoformans* alone. Percentage killing of *Cn* wild-type H99 by conditioned media collected from HL-60 cells undifferentiated (UD), differentiated (D) and from HL60 D treated with the PKD1 inhibitor benzoxoloazepinolone (CID755673). # *P*<0.05 compared with UD cells. * *P*<0.05, compared to untreated D cells.

Therefore, to test whether the release of antimicrobial factors by HL-60 cells is responsible for the killing of *C. neoformans*, 8×10^4^ HL-60 cells were differentiated, washed, incubated in fresh medium for 6 hours. The medium was then collected by centrifugation and combined with 4×10^3^ *C. neoformans* cells as above. As a control for killing, *C. neoformans* cells were incubated with the medium collected from undifferentiated cells. As a control for CFU, *C. neoformans* cells were incubated with fresh medium. CFU of *C. neoformans* in conditioned media (from differentiated or undifferentiated HL-60) were normalized with CFU of *C. neoformans* in fresh medium. It was found that conditioned media collected from differentiated HL-60 significantly killed *C. neoformans* cells (**Figure 4A**) in a similar pattern as observed when *C. neoformans* cells were incubated in the presence of HL-60 cells (compare **Figure 4A** with **Figure 1A**). Very interestingly, media collected from differentiated cells treated with D609 is no longer capable of killing *C. neoformans* (**Figure 4A**). These results strongly suggest that the killing activity of differentiated HL-60 might be due to some molecule(s) secreted in the medium and that this secretion may be controlled by SMS.

### Inhibition of Protein Kinase D (PKD) decreases the killing activity of differentiated HL-60

It has been shown that diacylglycerol (DAG) produced by SMS1 or SMS2 regulates the localization of PKD at the Golgi [30], and that the PKD at the Golgi, in turn, regulates vesicular trafficking and secretion. Thus we investigated the involvement of PKD in the extracellular killing activity of differentiated HL-60 cells. To this aim, we employed a specific PKD1 inhibitor, benzoxoloazepinolone (CID755673) that was recently identified and characterized [46]. Similarly to the experimental design in which SMS inhibitor was used (**Figure 4A**), different concentrations of the PKD1 inhibitor were incubated with differentiated HL-60, and after 6 hours of incubation the medium was collected and added to *C. neoformans* cells. It was found that treatment with 1 µM of CID755673 completely blocked the killing activity of the conditioned medium of differentiated HL-60 cells (**Figure 4B**). This inhibition was also observed when 5 and 10 µM CID755673 were used. As a control, CID755673 was tested for a possible effect on *C. neoformans* growth and HL-60 viability, and it was found that CFU of *C. neoformans* plus CID755673 were not different from CFU of *C. neoformans* minus CID755673 (**Figure 4B**). Also, CID755673 had no effect on HL-60 viability analyzed by Trypan blue exclusion (data not shown). These results suggest that PKD1 regulates the killing of *C. neoformans* by neutrophils through the secretion of an anti-cryptococcal factor(s) in the medium.

### Down-regulation of PKD1 but not PKD2 decreases killing of C. neoformans by differentiated HL-60 cells

**Figure 5:**
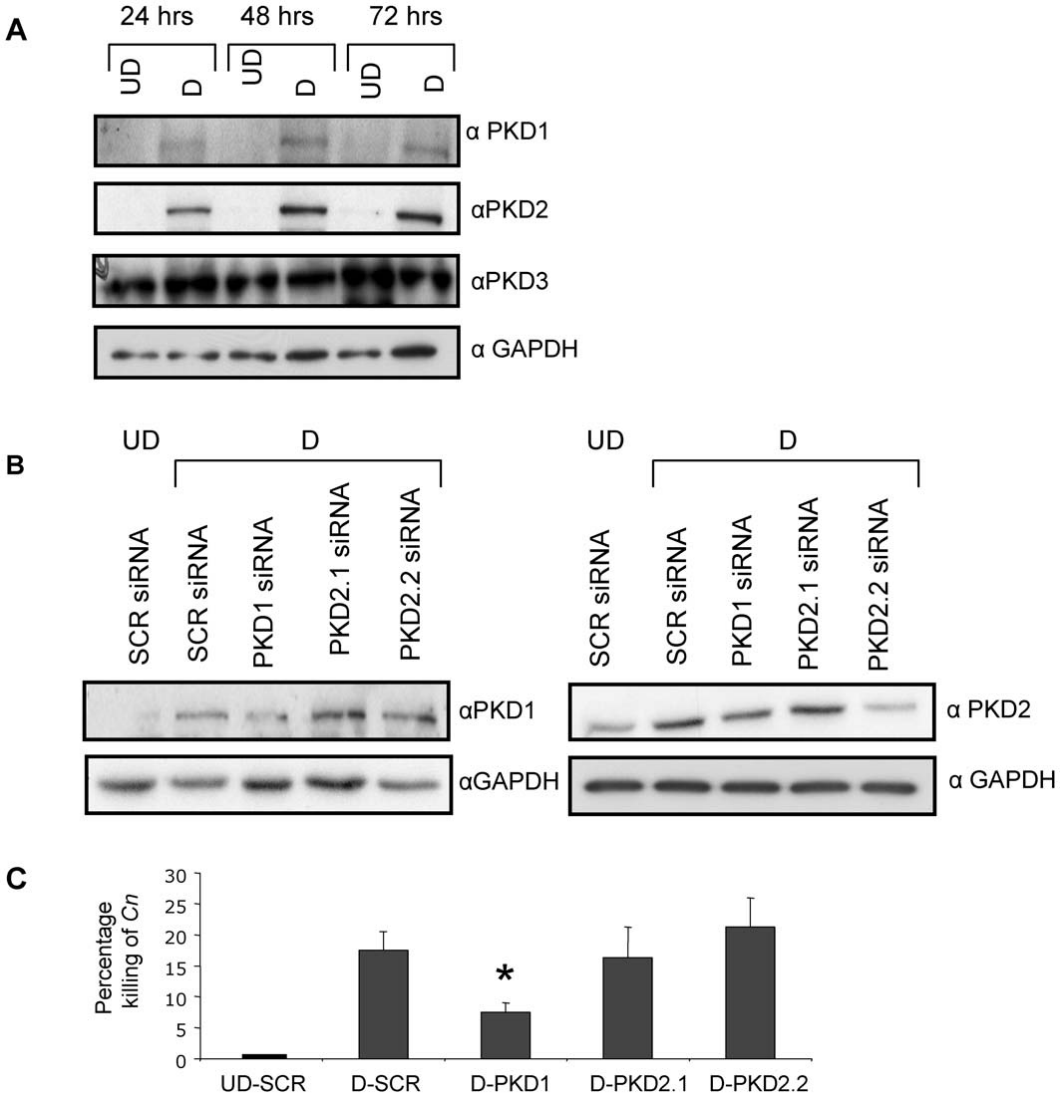
PKD isoforms during HL-60 cell differentiation. (**A**) HL-60 cells were plated at 1×10^5^ cells/ml and differentiation was induced. Differentiated and undifferentiated cells were collected at 24, 48 and 72 hrs and cellular lysates analyzed by Western blot for PKD1, PKD2, PKD3 and GAPDH. Blots are representative of 3 experiments. (**B**) Effect of modulation of either *PKD1* or *PKD2* by siRNA on PKD1 and PKD2 protein levels and GAPDH as loading control. (**C**) Effect of *PKDs* siRNA on the killing activity of differentiated HL-60 cells against *C. neoformans*. Results are representative of at least 3 independent experiments. * *P*<0.05, compared to D-SCR cells. UD, undifferentiated cells; D, differentiated cells; SCR: control scrambled siRNA.

Since the pharmacological inhibitor CID755673 inhibited killing of *C. neoformans*, we wanted to confirm this observation by specific down-regulation of PKD isoforms. Western Blotting (**Figure 5A**) analysis revealed that of the three PKD isoforms only PKD1 and PKD2 are expressed over the time course of differentiation whereas PKD3 protein levels remain unchanged. Therefore we sought to specifically down-regulate PKD1 and PKD2 in differentiated HL-60 cells to the levels observed in undifferentiated cells. To control for the efficacy and specificity of siRNA down-regulation, protein expression was analyzed by western blotting. As shown in **Figure 5B** **,** down-regulation of PKD1 did not affect PKD2 expression (**Figure 5B, left panel** and **Supplementary Figure S4**), and only the PKD2.2 siRNA sequence was effective in reducing PKD2 expression (**Figure 5B, right panel**) without reducing PKD1 levels. Therefore, to test the effect of specific down-regulation of PKD1 or PKD2 on the extracellular killing of *C. neoformans* by differentiated HL-60, down-regulated cells were incubated with *C. neoformans* for 4 h in a 20∶1 ratio (HL-60:*C. neoformans*), as described previously. The cultures were then collected and plated for fungal CFUs (**Figure 5C**). As a control for fungal growth, *C. neoformans* was incubated in the absence of HL-60 cells. The killing activity is represented by the percentage of *C. neoformans* CFU recovered from *C. neoformans* incubated alone minus the *C. neoformans* CFU recovered from *C. neoformans* cells incubated with differentiated HL-60 from each experimental group, divided by *C. neoformans* CFU recovered from *C. neoformans* incubated alone. It was found that only down-regulation of PKD1 and not PKD2 inhibited the killing of *C. neoformans* cells. These results provide further evidence that inhibition of PKD1 decreases the killing activity of differentiated HL-60 cells and that the specific isoform involved in regulating secretion of antifungal factor(s) must be PKD1. As observed with down-regulation of SMSs, down-regulation of PKDs did not impair differentiation of HL-60 cells (**Figure S2**).

### Diacylglycerol increases in differentiated compared to undifferentiated neutrophils

**Figure 6:**
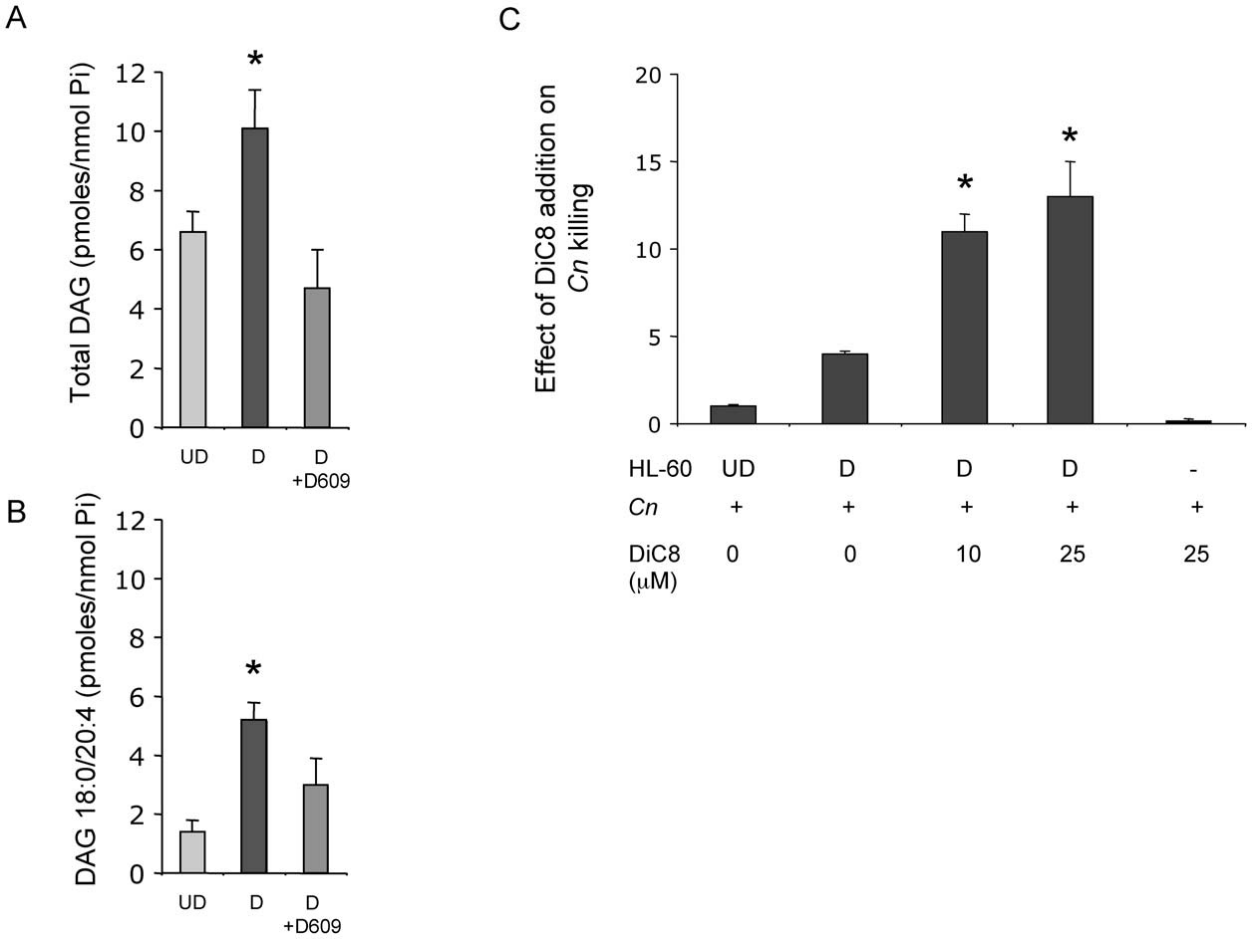
Mass spectrometry analysis of diacylglycerol in HL-60. (**A**) Total levels of DAG in HL-60 undifferentiated (UD), differentiated (D) and HL-60 D treated with D609, as measured by LC-MS and normalized by nanomole of lipid inorganic phosphate (Pi); * *P*<0.05, compared to UD cells. (**B**) Specific lipid species for DAG (18:0/20:4) in HL-60 undifferentiated (UD), differentiated (D) and HL-60 D treated with D609, as measured by LC-MS and normalized by Pi. * *P*<0.05, compared to UD cells. (**C**) Effect of DiC8 addition on killing activity of differentiated HL-60 cells against *C. neoformans* (*Cn*) or *C. neoformans* alone, represented as a fold response relative to HL-60 undifferentiated cells (UD). * *P*<0.05, compared to untreated D cells.

Since DAG produced by SMS1 or SMS2 regulates the localization of PKD at the Golgi [30], and SMS activity significantly increases in differentiated versus undifferentiated cells (**Figure 1B**), it was next examined by mass spectrometry whether DAG levels, as a result of SMS activity, would change in differentiated compared to undifferentiated cells. Total DAG levels increased in differentiated *versus* undifferentiated HL-60 cells (**Figure 6A**). In particular, it was found that DAG 18:0/20:4 was significantly up-regulated upon differentiation of HL-60 towards the granulocytic lineage (**Figure 6B**). We also found that two other DAG species (18:0/18:1 and Di C18:0) followed the same pattern observed for DAG 18:0/20:4 (data not shown). Other DAG species did not change significantly during differentiation (data not shown). These results suggest that, in differentiated HL-60 cells, the DAG profile supports the observed increase in SMS activity. We next examined the effect of exposure of differentiated HL-60 cells to exogenous DAG to see if killing effects were improved. To this aim, we employed 1,2-dioctanoyl-*sn*-glycerol (DiC8), a cell permeable analog of DAG. Similar to the experimental design in which SMS inhibitor was used (**Figure 1A**) different concentrations of DiC8 were incubated with differentiated HL-60, and after 2 hours of incubation *C. neoformans* cells were added. We found that treatment with 10 µM of DiC8 (dose adequate to mimic DAG-mediated effects in different cell lines; [30] improves the killing activity 11-fold compared to undifferentiated cells *versus* ∼2.5-fold for differentiated cells alone (**Figure 6C**). This effect is also observed when 25 µM is used. When used at less than 10 µM, DiC8 had no effect in differentiated HL-60 cells. As a control, DiC8 was tested for a possible effect on *C. neoformans* growth and HL-60 viability, and it was found that CFU of *C. neoformans* plus DiC8 were not different from CFU of *C. neoformans* minus DiC8 (**Figure 6C**). Also, DiC8 had no effect on HL-60 viability analyzed by Trypan blue exclusion (data not shown). These results support the involvement of DAG in the killing activity of HL-60 cells.

### Sphingomyelin increases in differentiated compared to undifferentiated neutrophils

**Figure 7:**
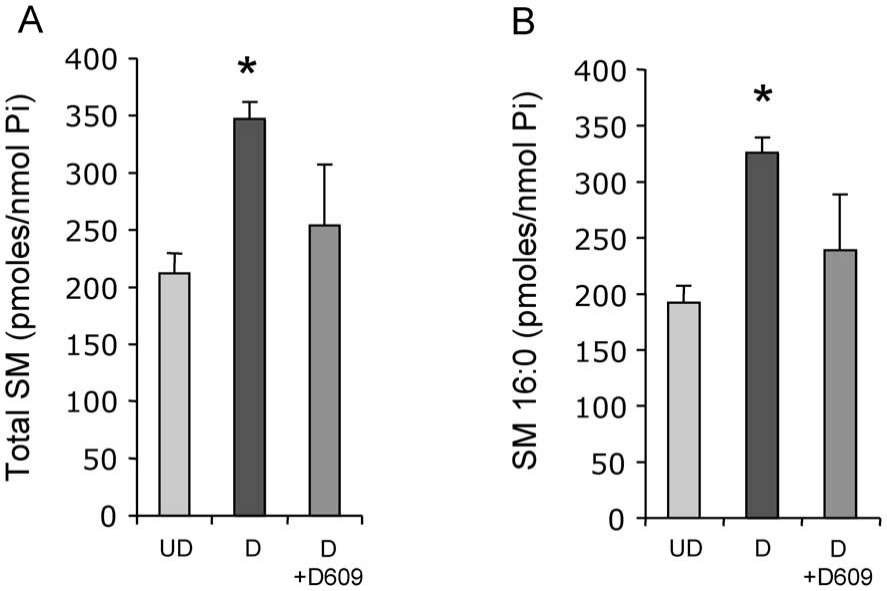
Mass spectrometry analysis of sphingomyelin in HL-60. (**A**) Total levels of SM in HL-60 undifferentiated (UD), differentiated (D) and HL-60 D treated with D609, as measured by LC-MS and normalized by nanomole of lipid inorganic phosphate (Pi). * *P*<0.05, compared to UD cells. (**B**) Specific lipid species for SM (16:0) in HL-60 undifferentiated (UD), differentiated (D) and HL-60 D treated with D609, as measured by LC-MS and normalized by Pi. * *P*<0.05, compared to UD cells.

Since sphingomyelin (SM) is also one of the lipids regulated by SMS, it was next examined by mass spectrometry whether SM levels, as a read out of SMS activity, would also change in differentiated *versus* undifferentiated cells. It was found that the total level of SM increased in differentiated compared to undifferentiated HL-60 cells (**Figure 7A**) and that this increase was due in particular to SM species 16:0 (characterized by the presence of palmitic acid attached to the sphingosine backbone) (**Figure 7B**). SM 16:0 is the most abundant SM species in HL-60 cells, followed by SM 24:1 [47] (which contains nervonic acid attached to the sphingosine backbone). SM 24∶1, as well as other minor SM species, did not significantly change between the HL-60 undifferentiated and differentiated phenotype (data not shown). On the other hand, the total level of ceramide, substrate of the SMS reaction, did not change significantly (**Figure S3A**) nor any of the species including ceramide C16:0 (**Figure S3B**). Overall, the observed lipid changes, in particular SM and DAG, support the enhanced SMS activity found in differentiated HL-60 cells.

### MALDI lung imaging during *C. neoformans* infection revealed a differential distribution of sphingomyelin (SM) species

Since fungal and mammalian cells produce similar species of DAG and ceramide, changes in these lipids cannot be used to study the role of SMS during *C. neoformans* infection. However, since SM is not produced by *C. neoformans*, its level in infected tissue will directly represent the level of activation of SMS (also only present in mammals and not in fungi). Since SM is the most abundant sphingolipid in membranes of mammalian cells, its high level allows a qualitative and quantitative assessment of SMS activity both in host cells and in tissues. Thus, to study whether SMS activity in the lung environment changes upon *C. neoformans* infection, measurements of SM were used as read out. To this aim, matrix-assisted laser desorption/ionization-mass spectrometric imaging (MALDI-MSI) was used. MALDI-MSI allows the visualization of the spatial distribution of specific molecules, according to their *m/z* ratio, within thin sections of tissue. The identification of the imaged molecule (*e.g.* lipid) was accomplished by MALDI tandem mass spectrometry where fragmentation patterns were compared to those of SM standards (see experimental procedures).

**Figure 8:**
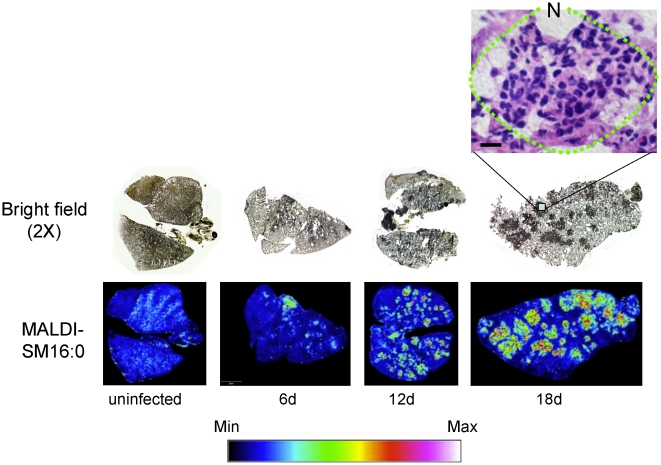
Matrix-assisted laser desorption-ionization mass spectrometry (MALDI-MS) of SM C16:0 in lung infected with *C. neoformans*. Lungs of CBA/J uninfected and infected mice with *C. neoformans* wild-type H99 strain were processed for MALDI tissue imaging and bright field photograph (2X) of a lung section stained with mucicarmine. Sphingomyelin (SM) 16:0 species ([M+Na]^+^ *m/z* 725) is concentrated in areas highly infiltrated with neutrophils, especially at 18 days of infection, as showed by hematoxylin and eosin staining at right. Min and Max, minimum and maximum intensity, respectively. N, neutrophils in dotted circle; black bar, 20 µm.

Since SM 16:0 was particularly elevated in the *in vitro* studies and this SM species is the most abundant in mammalian cells, we focused on the MALDI analysis of lung tissues of SM 16:0. Remarkably, there is a dramatic difference in the distribution of SM 16:0 species in *C. neoformans*-infected *versus* uninfected lung **(** **Figure 8** **)**. Whereas the signal associated with SM 16:0 is low and homogenously distributed in the uninfected lung, in lungs from infected mice it appears increased, clustered and more concentrated in specific areas and totally absent in other areas, especially at day 18 of infection. When the same lung was stained with hematoxylin and eosin (H&E), it was found that the areas in which SM is highly concentrated are also heavily infiltrated with neutrophils (**Figure 8**). This suggests that the different distribution of SM 16:0 may be due to the relocation/recruitment of the neutrophils at the site of the infection and perhaps to an increased production of this lipid by phagocytes.

**Figure 9:**
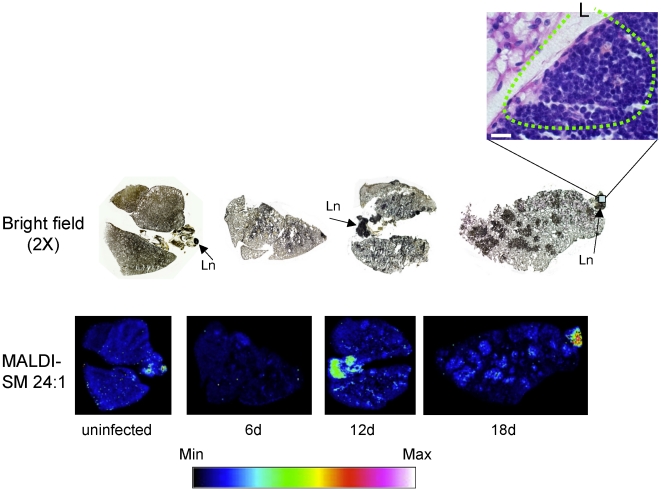
Matrix-assisted laser desorption-ionization mass spectrometry (MALDI-MS) of SM C24:1 in lung infected with *C. neoformans.* Lungs of CBA/J uninfected and infected mice with *C. neoformans* wild-type strain were assayed for MALDI tissue imaging and bright field photograph (2X) of a lung section stained with mucicarmine. Sphingomyelin (SM) 24∶1 ([M+Na]^+^ *m/z* 835) species is not concentrated in the lung but in hilar and apical lymph nodes, as showed by hematoxylin and eosin staining. (Ln, lymph node). Min and Max, minimum and maximum intensity, respectively. L, lymphocytes in dotted circle. White bar, 20 µm.

The second most abundant SM species in mammalian cells is SM 24∶1. Thus, SM 24∶1 was also analyzed by MALDI imaging. In contrast to SM 16:0, SM 24∶1 is distributed in a totally different manner (**Figure 9**). The signal associated with SM 24:1 is almost absent in lung tissues (infected or uninfected) and exclusively present in lymph nodes, in which lymphocytic cells predominate, as illustrated by the H&E staining (**Figure 9**). These results suggest that SM species are differentially represented in lung tissues. These results also suggest that different host immune cells may produce different species of SM; phagocytic cells seem to be specialized in producing SM 16:0 whereas lymphocytes may be specialized in producing SM 24:1.

### Lipid analysis by HPLC-MS/MS

**Figure 10:**
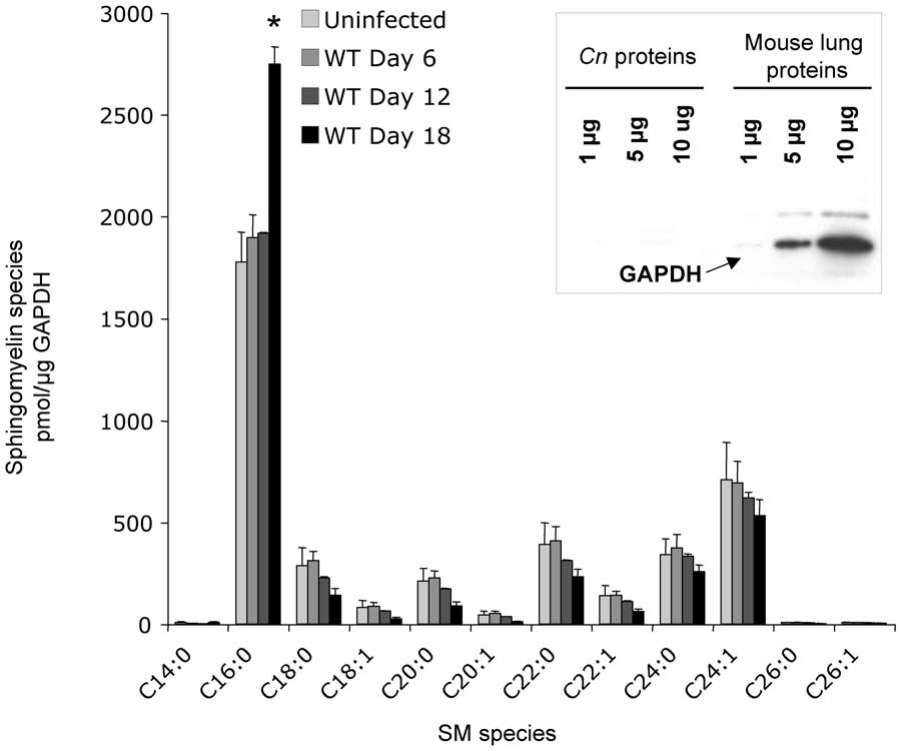
Liquid Chromatography Mass spectrometry (LC/MS) analysis of mouse lungs infected with wild-type *C neoformans* (*Cn*). Quantitative measurements by mass spectrometry (HPLC-MS/MS) of different sphingomyelin (SM) species in uninfected and Wild-type (WT)-infected lungs excised from CBA/J mice. Data were normalized to the level of mouse GAPDH protein in lungs using mammalian anti -glyceraldehyde 3-phosphate dehydrogenase (GAPDH) antibody from Ambion (Cat # AM4300), which shows specificity against the mammalian and not against the *Cn* GAPDH protein (inset). *, *P*<0.05, Day 18 versus Day 12.

While MALDI tissue imaging provides a sense of local expression levels, it does not provide a quantitative assessment of the total molecule(s) in the tissue. Therefore liquid chromatography-mass spectrometry (LC-MS) was performed in total lung homogenates and the level of different species of SM was measured. Before proceeding with the determination of the sphingolipid measurements in infected lungs, several pilot experiments were performed in uninfected mice to determine the best way to normalize the results. In fact, since SM is only present in mammalian cells, it was important to identify a factor that would be only expressed in mammalian cells and whose expression does not change during the course of the infection. This factor could be then used to normalize tissue SM level in both uninfected and infected lungs. Two mammalian proteins were considered for investigation: β-actin and glyceraldehyde 3-phosphate dehydrogenase (GAPDH). β-actin was excluded because anti-β-actin antibody (GenScript) cross-reacted against *C. neoformans* actin (data not shown). The antibody anti-GAPDH (Ambion) reacted against mammalian GAPDH only and did not cross-react with *C. neoformans* GAPDH or any other protein extracted from a *C. neoformans* cell culture (**inset in** **Figure 10**). Thus, a mammalian recombinant GAPDH obtained from Sigma was used to produce a standard curve, which then was used to determine the amount of GAPDH in uninfected and infected lungs at day 6, 12 and 18 days post infection (data not shown). It was found that the level of mouse GAPDH in lungs did not significantly change upon the challenge with *C. neoformans* (data not shown), indicating that this mammalian protein could successfully be used to normalize the lipid data.

Therefore, a different set of uninfected and infected lungs were separately homogenized, and an aliquot of the homogenate was used for the mass spectral data acquisition and another aliquot was used for the quantitation of GAPDH by Western blot using the LabWorks Image Acquisition and Analysis software from UVP BioImaging Systems, version 4.5. The results in **Figure 10** illustrate the detection of different species of SM, such as 16:0, 18:0, 18:1, 20:0, 20:1, 22:0, 22:1, 24:0, and 24:1. Among the measured species, SM 16:0 and 24:1 were the most abundant. This was not surprising because these two species have been shown to be the most abundant in many cellular types and tissues [47], [48], [49], [50], [51], [52], [53]. Importantly, LC-MS results showed that *only* SM 16:0 is significantly elevated at day 18 post infection compared to uninfected mice, suggesting an increased representation of this sphingolipid in the lung during the *C. neoformans* infection. We could not detect any SM 14:0, 26:0 and 26:1, suggesting that these species are either not produced by cells found in the mouse lung or that their levels are too low to be detected by MS. These data corroborate the results observed using MALDI imaging and point to phagocytic cells as the main producers of SM in lung infected by *C. neoformans*.

### Neutrophils kill *C. neoformans* cells and inhibition of both SMS activity and PKD totally abrogates the killing activity by neutrophils

**Figure 11:**
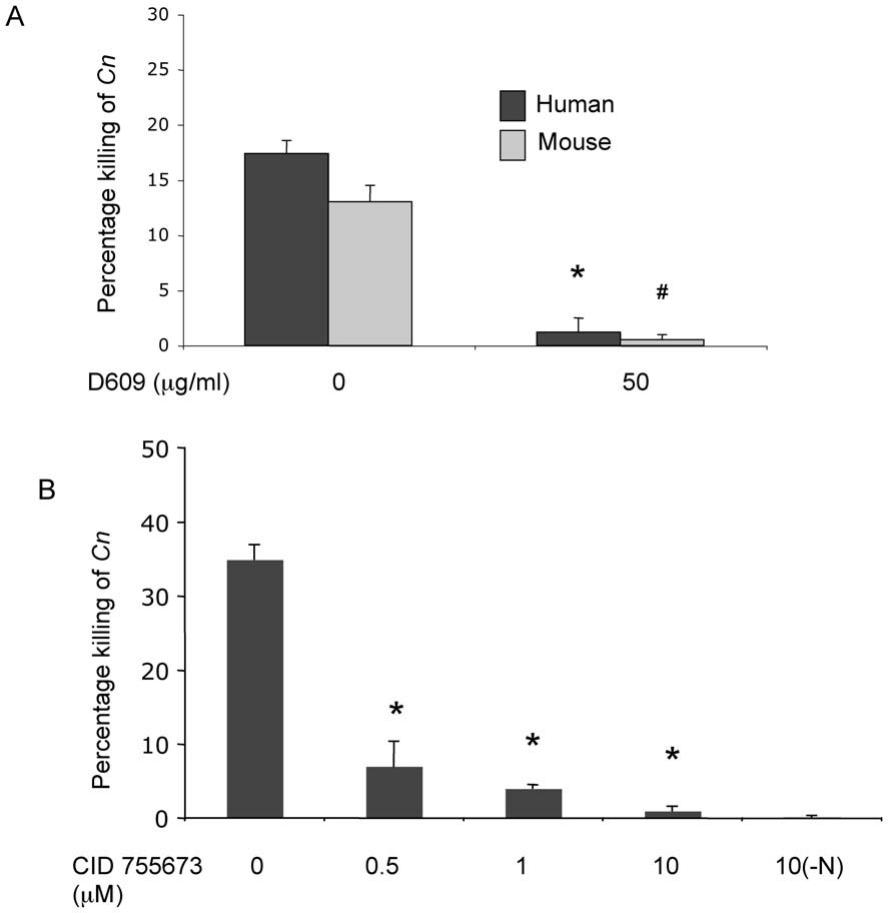
Effect of inhibitors on *C. neoformans* killing in the presence of neutrophils. (**A**) Effect of SMS inhibition on *C. neoformans* killing in the presence of neutrophils: 50 µg/ml D609 inhibits killing of *C. neoformans* in human and mouse neutrophils. *, *P*<0.05, treated versus human untreated cells; #, *P*<0.05, treated versus mouse untreated cells. (**B**) Effect of PKD1 inhibition (CID 755673) on killing of *C. neoformans* in the presence and in absence (-N) of human neutrophils. *, *P*<0.05, treated versus untreated cells.

In order to investigate the physiological relevance of our observations, we sought to investigate if neutrophils would be activated on exposure to *C. neoformans* cells and kill them. To this aim, we employed both fresh human normal peripheral blood-neutrophils and murine neutrophils and carried out the killing assay as described previously (**Figure 1A**). It was found that both human and mouse neutrophils kill *C. neoformans* and that the pharmacological SMS inhibitor protected *C. neoformans* from the killing activity of human and mouse neutrophils approximately 2-fold and 3-fold respectively (**Figure 11A**). Similarly, 0.2 µM of the PKD1 inhibitor CID755673 [reported IC_50_ for CID755673 [46]] protected the survival of *C. neoformans* during incubation with human neutrophils by a factor of almost 3 (**Figure 11B**). The difference in the effective CID755673 concentration between primary and cultured neutrophils (**Figure 4B**) may be attributable to relative levels and activities of endogenous PKDs [46].

## Discussion

In this investigation, the role of host sphingolipids during an infection caused by the fungal pathogen *C. neoformans* has been studied. Our results show that neutrophilic cells kill *C. neoformans* in an SMS-dependent manner. Furthermore, the killing activity of these cells is mainly due to secreted molecule(s) found in the medium and its release is dependent on PKD1 activity.

To investigate the relationship between SMS and its effect on killing of *C. neoformans* by differentiated HL-60 cells, we utilized two approaches: pharmacological and siRNA inhibition. D609, a pharmacological PC-PLC inhibitor, has previously been shown to inhibit SMS activity *in vitro* [27], [44], [54], and cellular studies showed that SMS activity was inhibited by concentrations used to previously study PC-PLC [43], [44], [55]. Those results showed that while it is thought that D609 may be non-specific, SMS *is* an important biochemical target for D609. The possibility that many of the roles of PC-PLC may be attributable to SMS cannot be ruled out, particularly as the PC-PLC gene has not been identified in mammalian cells [56]. In order to rule out any off-target effects of the inhibitor however, we also utilized a siRNA approach.

The use of siRNA to *SMS1* and *SMS2* allowed us to show that both SMSs play a role in the extracellular killing of *C. neoformans* by neutrophils. Interestingly, the depletion of either *SMS1* or *SMS2* by siRNA strongly inhibits the antimicrobial action of HL-60. One might have expected a partial effect but a total blockage may suggest that SMS activity at the Golgi upon siRNA reaches a low “threshold” level after which the antimicrobial activity of these cells is totally lost. The involvement of both SMSs in this process is not surprising as we propose that the SMS activity responsible for *C. neoformans* killing is the one present at the Golgi. Previous studies have shown that down-regulation of *SMS1* or *SMS2* reduces *de-novo* synthesis of SM each by 30–70% depending on the cell type [29], [30], [55]. Since *de-novo* synthesis of SM occurs only at the Golgi, these observations indicate that SMS1 and SMS2 show comparable activities in this organelle. As a consequence, the killing activity regulated by the SMS1-DAG-PKD1 pathway is comparable to the one regulated by SMS2-DAG-PKD1. On the other hand, SMS2 is also present at the plasma membrane [27], [30] but our results suggest that this pool may not be relevant to the antimicrobial action of neutrophils against *C. neoformans*. To our knowledge, this is the first evidence showing that SMS is directly involved in the regulation of the killing of a fungal organism by cultured and fresh neutrophils.

It is interesting to note that the killing activity of the medium is lower than the killing activity of the cells (compare **Figures 1A** and **4A**). The medium used in **Figure 4A** was obtained after 6 hours incubation of differentiated HL-60 cells in fresh medium, to reproduce the conditions used for the killing of *C. neoformans* by HL-60 cells illustrated in **Figure 1A**. Thus, the presence of any antifungal factor(s) in the medium (**Figure 4A**) is the result of constitutive secretion by differentiated cells that have not been exposed to *C. neoformans* cells. This secretion is clearly blocked by SMS and PKD1 inhibitors, suggesting a role for SMS-PKD1 pathway in this process. Interestingly, the killing activity of the differentiated cells (**Figure 1A**) is higher than that of the medium alone. Since in our experimental conditions, we have not observed intracellular killing of *C. neoformans* by differentiated HL-60, it is reasonable to hypothesize that the presence of *C. neoformans* cells may enhance the secretion of antifungal factors. In addition, since inhibition of SMS activity (either with D609 or siRNA) during the co-incubation of *C. neoformans* with differentiated HL-60 cells completely blocks the extracellular killing, it is proposed that SMS not only has a role in constitutive but also in the *C. neoformans-induced* secretion of antifungal factors.

In previous studies, we showed that DAG produced in the Golgi by SMS favors PKD1 translocation to this organelle [30] where it initiates trans Golgi network (TGN)-mediated secretion [57], [58] and we also observed that SMS1 and SMS2 modulate protein trafficking from the TGN to the plasma membrane and secretion (Marimuthu, Qureshi and Luberto, submitted). In the present study, we provide evidence of the involvement of SMS and specifically of PKD1 in the secretion of antimicrobial factors. In fact, treatment with benzoxoloazepinolone (CID755673), which at the concentrations used in this study specifically targets PKD1 [46], totally blocked the killing activity of both the media collected from differentiated HL-60 cells (**Figure 4B**), and from fresh human neutrophils (**Figure 11B**). In addition siRNA targeting PKD1, but not that targeting PKD2, inhibited the killing of *C. neoformans* (**Figure 5C**), supporting an isoform-specific function. So far, three PKD isoforms (PKD1, PKD2 and PKD3) have been described [59], [60], thus we also investigated the role of PKD3, in our system. Differently from PKD1 or PKD2, PKD3 level did not increase during differentiation, suggesting that its activity is not regulated during differentiation as PKD1 or PKD2. On the other hand, a role for PKD3 in secretion of antimicrobial factors cannot be ruled out. Although all PKDs are regulated by a DAG-binding C1 domain and by an activation loop, they have been shown to regulate secretion of different cargo molecules. For instance, PKD1 has been involved in secretion of matrix metalloproteinase-2 and -9 from prostate cancer cells [61] or of insulin [62]; PKD2 was shown to regulate hypoxia-induced VEGF-A secretion from pancreatic tumor cells [63] or secretion of chromogranin A from neuroendocrine tumor cells [64]; and PKD3 promoted secretion of cholecystokinin-mediated pancreatic amylase [65]. On the other hand, none of the PKDs has ever been linked to the release of antimicrobial peptides and/or regulation of infections, thus our studies are novel. Moreover, the fact that exposure of neutrophilic cells to *C. neoformans* may stimulate secretion and that this stimulated secretion is under the control of SMS activity suggests involvement of PKD1 activity not only in constitutive but also in regulated secretion, an area of investigation not thoroughly developed yet.

Since PKD1 is activated by DAG, the observed increase of DAG during differentiation of HL-60 cells (**Figure 6A and 6B**) in the background of elevated SMS activity (**Figure 1B**), supports the mechanism whereby SMS regulates PKD1 through the production of DAG. This is further supported by the fact that the exogenously added DAG analogue, DiC8 also improved the killing of *C. neoformans*. Interestingly, ceramide level does not change upon differentiation (**Figure S3**). This may suggest a compensatory mechanism by other sphingolipid metabolizing enzymes involved in the production of this sphingolipid [**66**].

Whereas DAG seems to be the bioactive mediator of SMS activity in this model, SM can be employed as a read-out for changes in SMS activity since SM significantly increases during differentiation (**Figure 7A, 7B**), in agreement with the observed increase in SMS activity (**Figure 1B**). In line with these observations, MALDI-MSI results clearly show that primary neutrophils are loaded with SM C16:0 similarly to the cell culture model (differentiated HL-60), and thus SM captured by this technique can be considered as a spatial read out for SMS activity in tissue section. These results are even more significant because, in contrast to DAG, SM is the only mammalian specific component of the SMS reaction and it is not present in *C. neoformans* cells. Thus, host SM level and distribution can be unequivocally determined in tissues infected with *C. neoformans*.

Interestingly, phagocytic cells seem specialized to produce SM 16:0, whereas lymphocytes may produce SM 24:1. Using classical histology analysis, we observed that the areas in which SM 16:0 is highly concentrated are heavily infiltrated with neutrophils, which increase over the duration of infection. This distribution of SM 16:0 over time may be due either to the relocation of resident lung neutrophils at the site of infection and/or recruitment of additional phagocytes to the site. Since LC-MS analysis of SM species reveals a net increase of SM 16:0 in the lungs in the course of the infection, it is possible that along with the re-distribution of phagocytes there might be also a stimulation of SM synthesis. On the other hand, it is possible that the increase of SM 16:0 levels might be associated with the recruitment of phagocytes from the bloodstream to the lung. To our knowledge, this is the first time that the MALDI-MS imaging technology has been used to examine how host sphingolipids are modulated during an infection.

We cannot rule out that the increase of SM at the site of infection may contribute to the antifungal activity by phagocytic cells *in vivo*. In fact, it has been shown that SM itself can enhance NFκB activation [33], and NFκB is a transcription factor and a well-known regulator of a variety of host immune cellular responses against infection [39]. In addition, receptor-mediated NFκB activation, and its target gene expression, is impaired in sms2-/- knockout mice [38], suggesting that the regulation of the antifungal action by SMS may involve the regulation of gene transcription in addition to protein secretion. DAG can also activate NFκB [33], [45] and thus, DAG and SM can act synergistically in promoting the killing activity of fungi by neutrophils. These possibilities may have an impact during the infection in the animal where interaction between different immune cells is important to control *C. neoformans*. On the other hand, it is not a likely scenario in the *in vitro* system, in which the differentiated HL-60 cells are the only player against *C. neoformans*. Given the antifungal activity of SMS in neutrophils, an up-regulation of SMS activity (*e.g.* by lentiviral expression) in these cells may enhance their killing activity towards microorganisms that cause infection. This strategy could be employed to replenish the killing activity of these cells, especially in conditions of immunodeficiency in which the antimicrobial activity of resident neutrophils could be impaired.

Studies on host defense against *C. neoformans* emphasize the critical role of T cell immunity for containment of *C. neoformans* infection through the activation of macrophages and the recruitment of neutrophils resulting in granuloma formation in healthy individuals (Reviewed in [10], [11], [12]). Although macrophages are considered the first line of defense against *C. neoformans*, the role of neutrophils is equally important because, once recruited, they are extremely efficient in killing *C. neoformans* and other fungal cells [21], [22]. Indeed, macrophages are known to kill microorganisms mainly by phagocytosis whereas neutrophils, beside phagocytosis, also kill extracellularly, as extensively discussed in a recent review by Silva [67]. On the other hand, previous studies in mice have shown that neutropenia via antibody depletion resulted in no effect on *C. neoformans* virulence in i.v. infection whereas with i.n. infection neutropenic mice actually survived longer [68]. These results could simply be related to the inability of mouse neutrophils to produce α-defensin [69]. Thus, studies in transgenic mice in which neutrophils are expression human α-defensin are warranted to understand the precise contribution of these immune cells to the host fight against *C. neoformans* and, eventually, to identify the molecular modulator(s) of their immune activity. This is important because the possibility to use modified human neutrophils as a mean to combat fungal infections has been proposed against *Ca* [23], [24], [70] and it could be exploited also against *C. neoformans*.

The identity of the antimicrobial factors present in the medium and responsible for the killing of *C. neoformans* cells by neutrophils is currently unknown. Neutrophils produce numerous antimicrobial granules and enzymes over the course of differentiation and release them extracellularly [21], [22] [71]. Among these antimicrobial factors, defensins and myeloperoxidase (MPO) seem to be the primary candidates in our experimental model. In fact, defensins have been shown to be extremely cytotoxic towards *C. neoformans* [72], [73] and mice lacking MPO are hyper-susceptible to *C. neoformans* [74], [75] and other fungal infections [74], [76], [77]. Thus, in future experimentation, it is our intension to identify the antifungal factors under the control of the SMS-PKD1 pathway starting with these candidates.

In conclusion, these studies indicate a key role for SMS in the regulation of the killing activity of neutrophils against *C. neoformans* and this killing activity appears to be regulated by the lipid, in particular DAG, produced by SMS. We also propose that the mechanism by which SMS mediates the killing by neutrophils is through modulation of extracellular release of antifungal factor(s) through a PKD1-mediated mechanism. These studies reveal a novel role for the host sphingolipid pathway in the regulation of the infection caused by *C. neoformans*, and provide new insights into the mechanisms by which host sphingolipids control infections. These mechanisms could be exploited for the development of novel therapeutic strategies.

## Supporting Information

Figure S1 Changes in the mRNA levels of SMS isoforms during HL‐60 cell differentiation induced by DMSO and retinoic acid. HL‐60 cells were plated at 1×10^5^ cells/ml and differentiation induced by treating with 1.3% DMSO and 2.5 µM retinoic acid. Undifferentiated cells received only vehicle solution for retinoic acid. Differentiated and undifferentiated cells were collected at 24, 48 and 72 hrs, total RNA was extracted, and RT‐PCR was performed using specific primers for *SMS1* (**A**) or *SMS2* (**B**) and *GAPDH*. The RT‐PCR results were analysed using Q‐gene software, which expresses data as the means of normalized expression. Results are representative of at least 3 independent experiments, and error bars represent SD and *P<0.05 compared with respective undifferentiated cells.
(TIF)

Figure S2 Effect of modulation of either PKD 1 or PKD 2 on HL‐60 cell differentiation. Two million HL‐60 cells were transfected with 4.5 µg of SCR, *PKD1* siRNA, *PKD2* (PKD2.2) siRNA by nucleofection. Differentiation was induced with DMSO and RA. Cells were collected at 48 hours and processed for flow cytometry analysis of CD11b positive cells. Results are representative of at least 3 independent experiments, and error bars represent SD and *P<0.05 compared with SCR undifferentiated cells. UD, undifferentiated; D, differentiated cells.
(TIF)

Figure S3 Mass spectrometry analysis of ceramide in HL‐60. (**A**) Total levels of ceramide, in HL‐60 undifferentiated (UD), differentiated (D) and HL‐60 D treated with D609, as measured by LC‐MS and normalized by nanomole of lipid inorganic phosphate (Pi). (**B**) Specific lipid species for ceramide (16:0) in HL‐60 undifferentiated (UD), differentiated (D) and HL‐60 D treated with D609, as measured by LC‐MS and normalized by Pi. *P<0.05 compared UD cells.
(TIF)

Figure S4 Quantitative analysis of Western blot of Pkd1 in Figure 5B. Intensity of the bands were calculated using LabWork Software. AU, arbitrary units.
(TIF)
