# Supplementary figures and images for: Role of Sphingomyelin Synthase in Controlling the Antimicrobial Activity of Neutrophils against Cryptococcus neoformans
Source: PLoS One. 2010 Dec 28;5(12):e15587. doi: 10.1371/journal.pone.0015587 (PMC3011003; doi:10.1371/journal.pone.0015587)

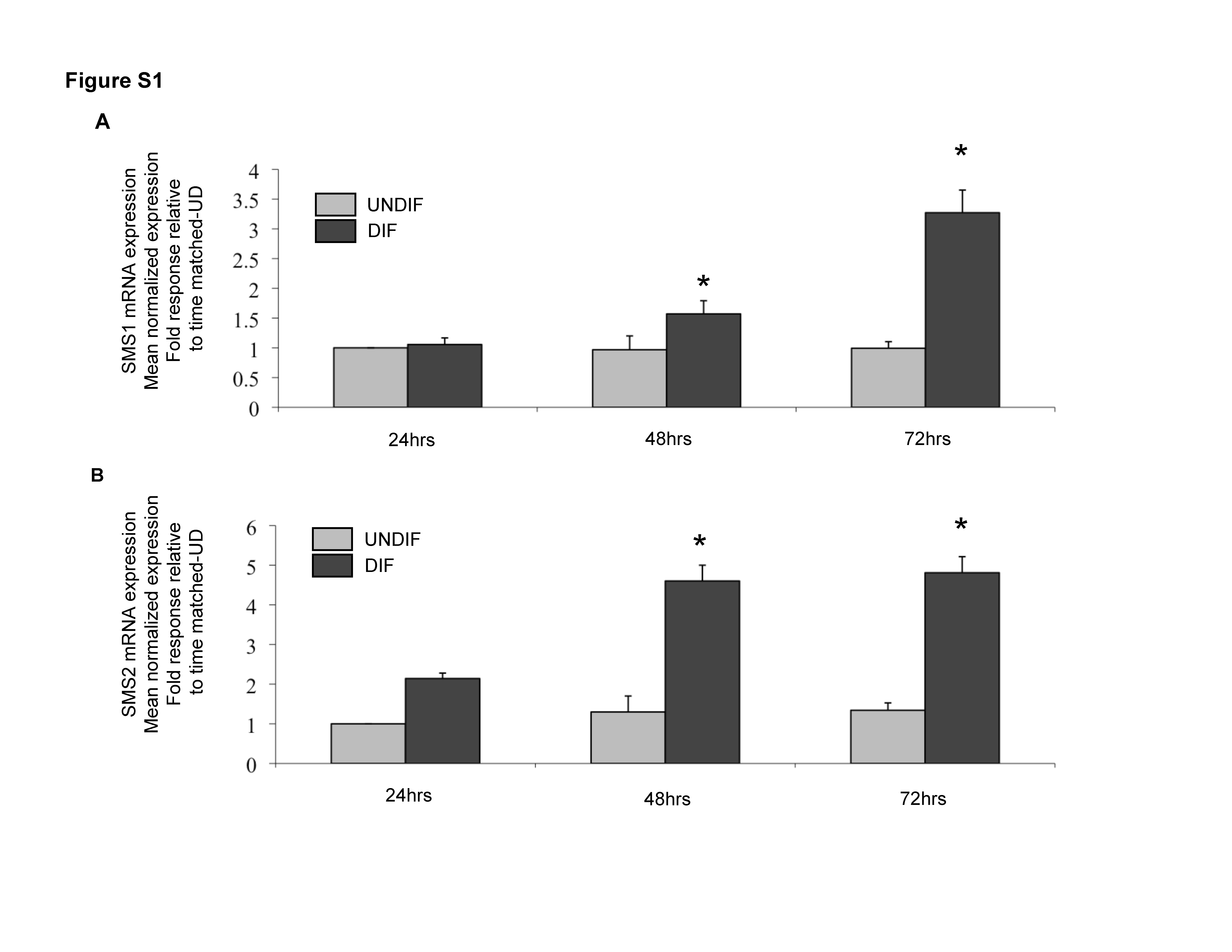

Supplement: Figure S1 — Changes in the mRNA levels of SMS isoforms during HL‐60 cell differentiation induced by DMSO and retinoic acid. HL‐60 cells were plated at 1×10^5^ cells/ml and differentiation induced by treating with 1.3% DMSO and 2.5 µM retinoic acid. Undifferentiated cells received only vehicle solution for retinoic acid. Differentiated and undifferentiated cells were collected at 24, 48 and 72 hrs, total RNA was extracted, and RT‐PCR was performed using specific primers for SMS1 (A) or SMS2 (B) and GAPDH. The RT‐PCR results were analysed using Q‐gene software, which expresses data as the means of normalized expression. Results are representative of at least 3 independent experiments, and error bars represent SD and *P<0.05 compared with respective undifferentiated cells. (TIF) [file pone.0015587.s001.tif]

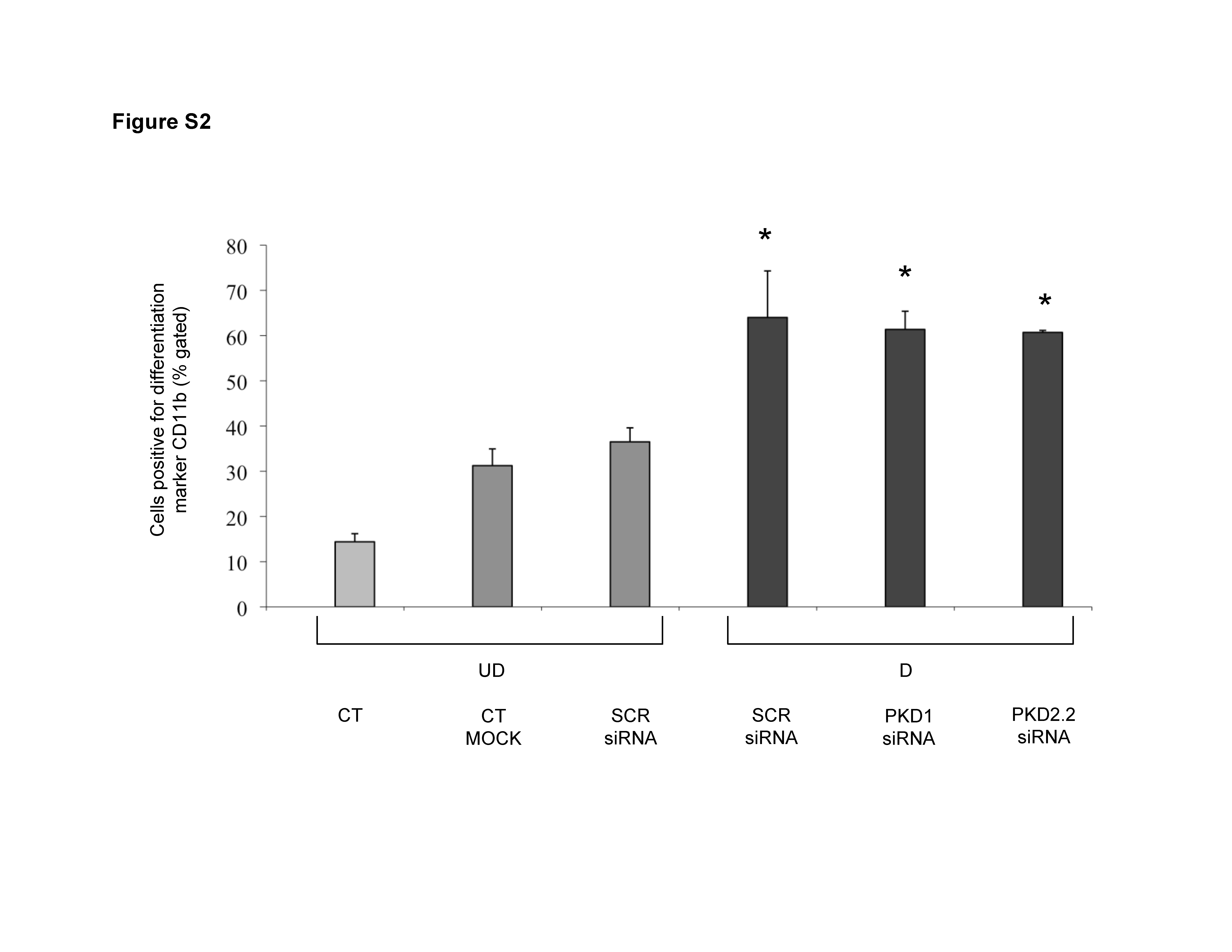

Supplement: Figure S2 — Effect of modulation of either PKD 1 or PKD 2 on HL‐60 cell differentiation. Two million HL‐60 cells were transfected with 4.5 µg of SCR, PKD1 siRNA, PKD2 (PKD2.2) siRNA by nucleofection. Differentiation was induced with DMSO and RA. Cells were collected at 48 hours and processed for flow cytometry analysis of CD11b positive cells. Results are representative of at least 3 independent experiments, and error bars represent SD and *P<0.05 compared with SCR undifferentiated cells. UD, undifferentiated; D, differentiated cells. (TIF) [file pone.0015587.s002.tif]

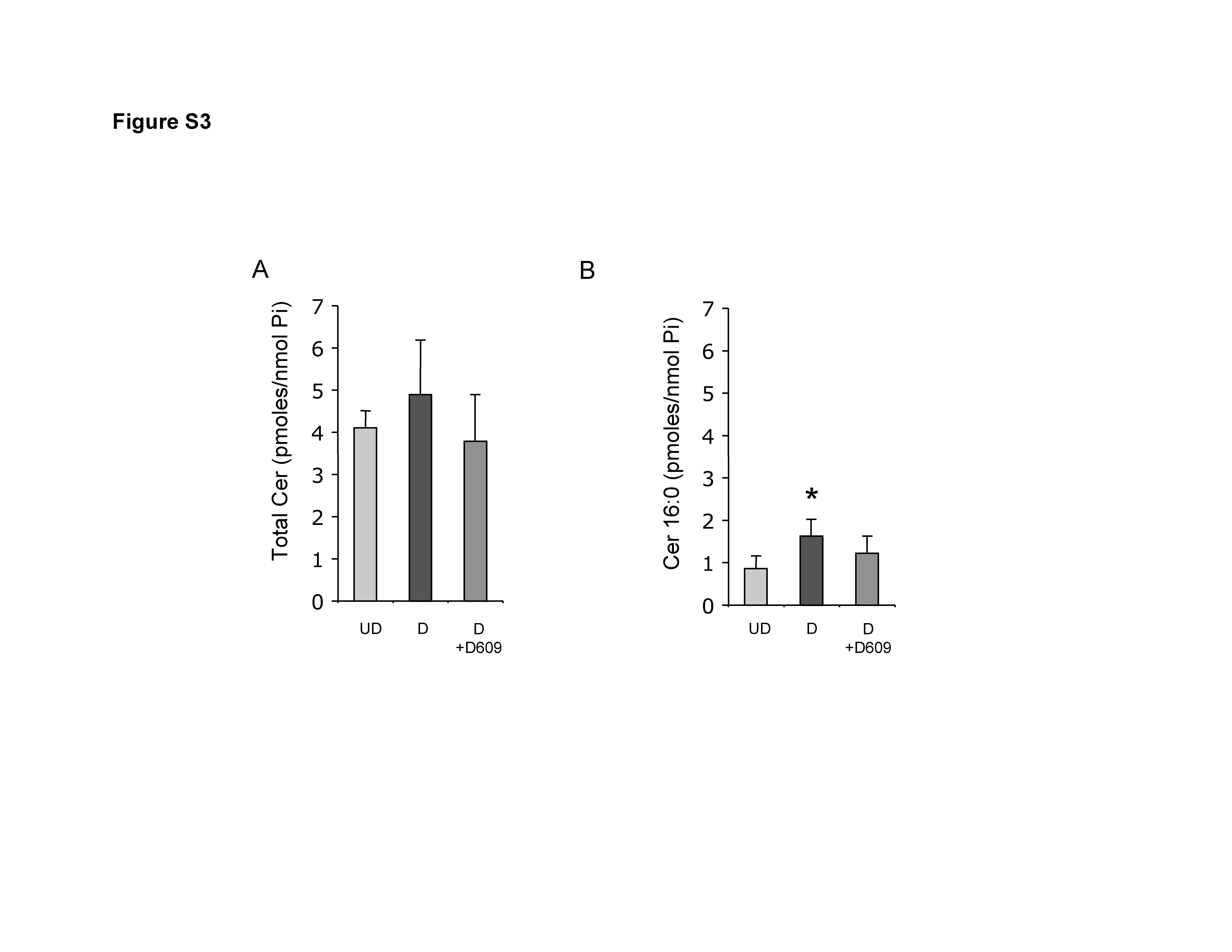

Supplement: Figure S3 — Mass spectrometry analysis of ceramide in HL‐60. (A) Total levels of ceramide, in HL‐60 undifferentiated (UD), differentiated (D) and HL‐60 D treated with D609, as measured by LC‐MS and normalized by nanomole of lipid inorganic phosphate (Pi). (B) Specific lipid species for ceramide (16:0) in HL‐60 undifferentiated (UD), differentiated (D) and HL‐60 D treated with D609, as measured by LC‐MS and normalized by Pi. *P<0.05 compared UD cells. (TIF) [file pone.0015587.s003.tif]

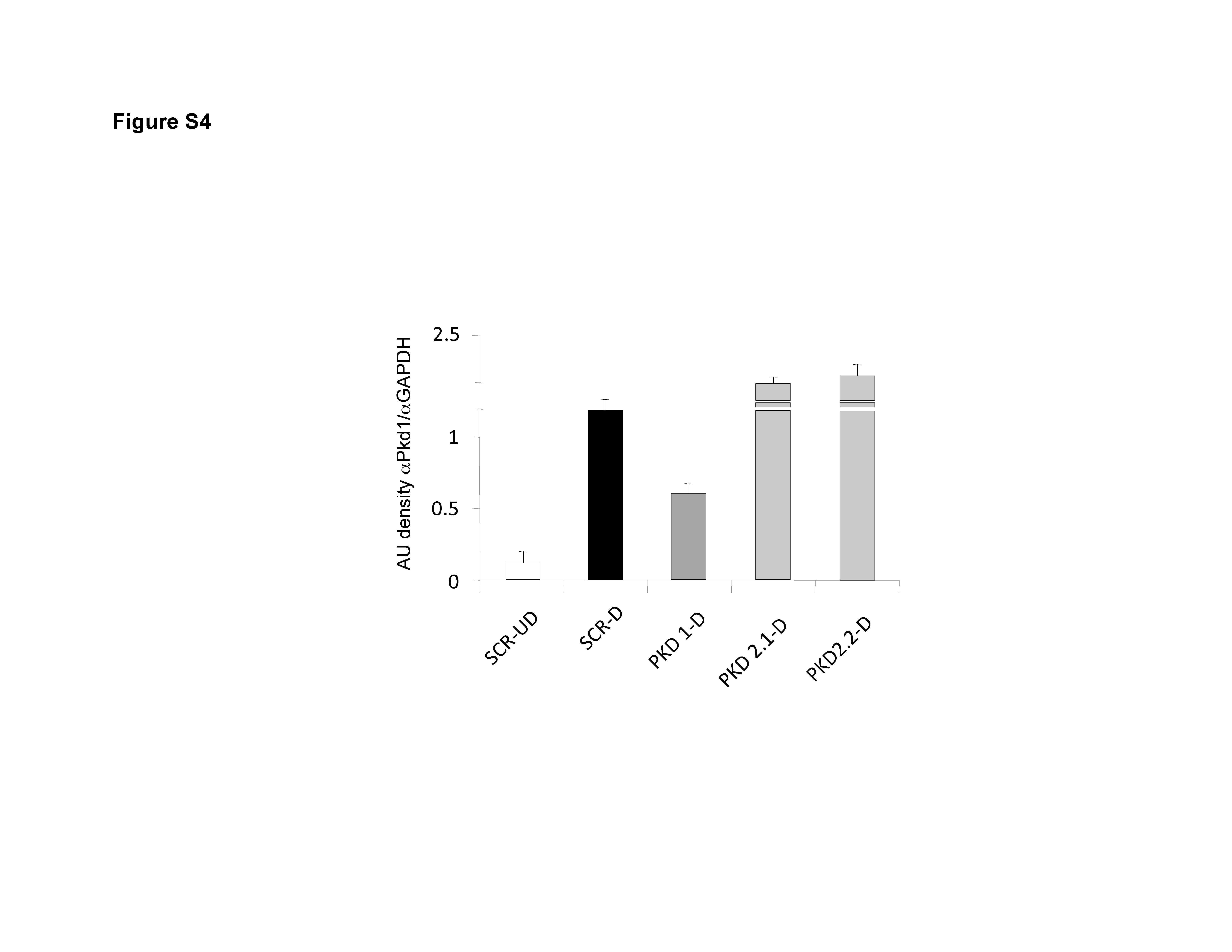

Supplement: Figure S4 — Quantitative analysis of Western blot of Pkd1 in Figure 5B. Intensity of the bands were calculated using LabWork Software. AU, arbitrary units. (TIF) [file pone.0015587.s004.tif]
